# A Bayesian random regression method using mixture priors for genome‐enabled analysis of time‐series high‐throughput phenotyping data

**DOI:** 10.1002/tpg2.20228

**Published:** 2022-07-29

**Authors:** Jiayi Qu, Gota Morota, Hao Cheng

**Affiliations:** ^1^ Dep. of Animal Science Univ. of California Davis Davis CA 95616 USA; ^2^ Dep. of Animal and Poultry Sciences Virginia Polytechnic Institute and State Univ. Blacksburg VA 24061 USA

## Abstract

The recent advancement in image‐based phenotyping platforms enables the acquisition of large‐scale nondestructive crop phenotypes measured at frequent intervals. To further understand the underlying genetic basis over a physiological process and improve plant breeding programs, the question of how to efficiently utilize these time‐series measurements in genome‐enabled analysis including genomic prediction and genome‐wide association studies (GWASs) should be considered. In this paper, a Bayesian random regression model with mixture priors is developed to introduce more meaningful biological assumptions to the analysis of longitudinal traits. The mixture prior for marker effects in Bayes *C*π is implemented in our developed model (RR‐BayesC) for demonstration purpose. The estimation of single‐nucleotide polymorphism–specific effects that are related to the dynamic performance of crops and the accuracy of genomic prediction by RR‐BayesC were studied through both simulated and real rice (Oryza sativa L.) data. For genomic prediction, three predictive scenarios were studied. In the simulated study, RR‐BayesC showed a significantly higher prediction accuracy than that obtained by single‐trait analysis, especially for days when heritability were low. In real data analysis, RR‐BayesC showed relatively high prediction accuracy when forecast is required for phenotypes at later period (e.g., from 0.94 to 0.98 for lines with observations at an earlier period and from 0.65 to 0.67 for lines without any observations). For GWASs, inference of single markers and inference of genomic windows were conducted. In the simulated study, RR‐BayesC showed its promising ability to distinguish quantitative trait loci (QTL) that are invariant to temporal covariates and QTL that interact with time. An association study of real data was also presented to demonstrate the application of RR‐BayesC in real data analysis. In this paper, we develop a Bayesian random regression model that is able to incorporate mixture priors to marker effects and show improved performance of genomic prediction and GWASs for longitudinal data analysis based on both simulated and real data. The software tool JWAS offers routines to perform our proposed random regression analysis.

## Core Ideas

- Large‐scale longitudinal data are obtained from high‐throughput phenotyping platforms.
- We developed a Bayesian random regression model with mixture priors to analyze such data.
- Our method is able to detect QTL that are invariant to or that interact with time.
- Our proposed method can forecast future phenotypes with improved prediction accuracy.

**Abbreviations**
CV: cross validation
GBLUP: genomic best linear unbiased prediction
GWAS: genome‐wide association study
MCMC: Markov chain Monte Carlo
MEM: marker effect model
pAUC: partial area under the receiver operating characteristic curve
PIP: posterior inclusion probability
PSA: projected shoot area
QTL: quantitative trait loci
RDP1: Rice Diversity Panel 1
RR‐MEM: random regression marker effect model
RRM: random regression model
SNP: single‐nucleotide polymorphism
WPPA: window posterior probability of association

## 1 INTRODUCTION

The acquisition of large‐scale nondestructive phenotypes (e.g., the combination of color infrared and red–green–blue images describing crop growth and biomass production) is possible due to the advancement in modern phenomics platforms (Mir et al., 2019). The current and future developments in the field of phenomics are expected to accelerate the breeding process and improve the efficiency of genomic prediction (Shakoor et al., 2017). Some widely implemented strategies are to use greenhouse‐based high‐throughput phenotyping or unoccupied aerial platforms to gather images of crops at frequent intervals throughout growth stages. To increase the biological understanding and shorten the duration of breeding cycles, it is important to answer the question of how to efficiently utilize these time‐series measurements, or longitudinal data, in genome‐enabled analysis, including genomic prediction and genome‐wide association studies (GWASs).

The random regression model (RRM) is a prevalent statistical model to analyze the continuous performance of individuals over time and to investigate the temporal variations through biological processes. The RRM was introduced by Henderson (1982) and Laird and Ware (1982) for the analyses of longitudinal data in animal breeding. Areas of RRM applications include lactation performance of dairy animals, growth curves of different species, survival analyses, and genotype by environment (G × E) interaction studies (Brito et al., 2017; Koivula et al., 2015; Rutten et al., 2005). The number of studies of RRM has increased over the past several years as high‐throughput phenomics emerges. In the RRM, given the relationship between observations and time, a continuous function of time could be used to capture the phenotypic trajectory. With the determination of phenotypic trajectory, random regressions are used to investigate the temporal individual variations caused by additive genetic effects and permanent environmental effects.

In the area of genomic prediction, genomic best linear unbiased prediction (GBLUP) is a prevalent model that is used to predict the genetic merit of individuals (Habier et al., 2007; VanRaden, 2008). In GBLUP, a genomic relationship matrix that describes genetic similarities between individuals computed from marker information is used to account for the covariances between individual additive genetic effects. The GBLUP model has been expanded to accommodate the analysis of longitudinal data, in which temporal additive genetic effects are estimated by incorporating the genomic relationship matrix into the RRM (hereinafter referred to as “RR‐GBLUP”) (Baba et al., 2020; Campbell et al., 2018). In addition to genomic prediction, the RRM enables GWAS for longitudinal traits (Campbell et al., 2019; Oliveira, Brito, et al., 2019), in which single‐nucleotide polymorphism (SNP)‐specific effects are obtained by backsolving additive genetic random regression coefficients to marker effects (Campbell et al., 2019; Oliveira, Lourenco, et al., 2019).

The GBLUP model can be reparameterized as a multiple‐regression model that simultaneously fits all markers as random effects, which is hereafter referred to as marker effect model (MEM). It has been shown that, when the marker effects follow a univariate normal distribution, MEM is equivalent to GBLUP in prediction of breeding values (Habier et al., 2007; Meuwissen et al., 2001; Whittaker et al., 2000). In the past few decades, a collection of Bayesian Alphabet models (Erbe et al., 2012; Gianola & Fernando, 2020; Habier et al., 2011; Kizilkaya et al., 2010; Meuwissen et al., 2001; Moser et al., 2015; Park & Casella, 2008) have been developed for genomic prediction and GWASs. Bayesian variable selection regression models such as BayesB and BayesC are widely used Bayesian Alphabet methods, in which mixture priors are used for marker effects. A major benefit of Bayesian variable selection regression models is that markers can be efficiently selected using Markov chain Monte Carlo (MCMC) methods. Furthermore, Bayesian regression models incorporating mixture priors have been illustrated to provide better performance than conventional GBLUP methods given efficient variable selection process and reasonable biological prior assumptions (Mehrban et al., 2017; Wolc et al., 2016). However, the use of Bayesian variable selection methods was not considered for genome‐enabled analysis of longitudinal traits derived from high‐throughput phenomics.

In this paper, we extend Bayesian variable selection regression models to incorporate RRM for the analysis of longitudinal data (hereafter referred to as the random regression marker effect model [RR‐MEM]). In the RR‐MEM, SNP‐specific effects are expressed as regression functions of temporal covariates, and mixture priors are assigned to regression coefficients of each marker. In this paper, the mixture prior in multi‐trait BayesC (Cheng et al., 2018) is used, such that regression coefficients of each marker are considered correlated, and each regression coefficient is allowed to have a null effect. Use of RR‐MEM with other priors, such as those in Bayesian LASSO (Gianola & Fernando, 2020; Park & Casella, 2008), should be straightforward. Our model is implemented in a software tool called “JWAS” (Cheng et al., 2018) to perform these analyses.

## 2 MATERIALS AND METHODS

### 2.1 Random regression models

The basic structure of RRM, in which the group is considered as the only fixed effect for simplicity, can be written as (Schaeffer, 2004):

(1) $$y_{ikt}=\sum_{l=0}^{m_{b}}b_{kl}x_{klt}+\sum_{l=0}^{m_{a}}a_{il}z_{a_{ilt}}+\sum_{l=0}^{m_{p}}p_{il}z_{p_{ilt}}+e_{ikt}$$

where *y_ikt_* is the observation on the *i*th individual at time point *t* belonging to the *k*th group; $\sum_{l=0}^{m_{b}}b_{kl}\;\;x_{klt}$is the fixed regression that depicts the phenotypic trajectory of observations across individuals belonging to group *k*; *m*_b_ is the order of the fixed regression function; *b_kl_* is the (*l* + 1)th fixed regression coefficient for group *k*; $\sum_{l=0}^{m_{a}}a_{\mathrm{il}}z_{a_{\mathrm{ilt}}}$accounts for the individual additive genetic effect, with *m*_a_ being the order of the random regression function; and $a_{il}$ is the (*l* + 1)th random regression coefficient for *i*th individual. In addition to the additive genetic effect, another random regression $\sum_{l=0}^{m_{p}}p_{il}z_{p_{ilt}}$is fitted in RRM to account for the permanent environmental effect for individual *i*, *e_ikt_* is the residual effect, and heterogeneous residual variance is usually assumed over different time points. The terms $x_{.lt}$, $z_{a_{.lt}}$, and $z_{p_{.lt}}$are regression covariates that allocate time *t* to the (*l* + 1)th fixed regression coefficients, additive genetic random regression coefficients, and permanent environmental random regression coefficients, respectively. For example, if a quadratic regression function of time is used to depict the phenotypic trajectory of group *k*, *x_klt_* is equivalent to 1,*t*,*t* ^2^ for *l* = 0,1,2, respectively. In most of the recent RRM applications (da Gama et al., 2021; Lázaro et al., 2021; Ogawa & Satoh, 2021), orthogonal polynomials, such as Legendre polynomials (Kirkpatrick et al., 1990), of standardized time points are used for random regressions due to their advantages in reducing correlations among estimated coefficients (Schaeffer, 2004).

In matrix notation, we assume same orders of polynomial functions (i.e., *m*_b_ = *m*_a_ = *m*_p_) for simplicity of our description and write RRM for observations of individual *i* across time points as

(2) $$y_{i}=X_{i(t)}b_{k}+Z_{a_{i}\left(t\right)}a_{i}+Z_{p_{i}\left(t\right)}p_{i}+e_{i}$$

where **y** *_i_* is a vector of length *t_i_* of phenotypes measured at *t_i_* time points for individual *i*; X*_i_*_(_ *_t_*_)_, $Z_{a_{i}\left(t\right)}$, and $Z_{p_{i}\left(t\right)}$ are matrices with elements in column *d* representing the *d*th regression covariates corresponding to the *t_i_* time points; **b** *_k_* is a vector of length (*m*_b_ + 1) of fixed regression coefficients that used to depict the phenotypic trajectory of group *k*; $a_{i}$ is a vector of length (*m*_a_ + 1) of additive genetic random regression coefficients for individual *i*; **p** *_i_* is a vector of length (*m*_p_ + 1) of permanent environmental random regression coefficients for individual *i*; and **e** *_i_* is a vector of length *t_i_* of residuals. For the random effects, it is assumed that

(3) $$\left(\begin{array}{c} a_{i}\\ p_{i}\\ e_{i} \end{array}\right)∼N\left(\left[\begin{array}{c} 0\\ 0\\ 0 \end{array}\right],\left[\begin{array}{ccc} K_{g} & 0 & 0\\ 0 & K_{\mathrm{pe}} & 0\\ 0 & 0 & R \end{array}\right]\right)$$

where **K**_g_ and **K**_pe_ are the (co)variance matrices of the additive genetic and permanent environmental random regression coefficients, respectively, and **R** is the (co)variance matrix of the residuals across the *t_i_* time points.

**Core Ideas**

- Large‐scale longitudinal data are obtained from high‐throughput phenotyping platforms.
- We developed a Bayesian random regression model with mixture priors to analyze such data.
- Our method is able to detect QTL that are invariant to or that interact with time.
- Our proposed method can forecast future phenotypes with improved prediction accuracy.

If the numerator relationship matrix, **A**, is used to depict the covariances among individuals' additive genetic effects as in Schaeffer (2004), the variance‐covariance structure of the additive genetic random regression coefficients for all individuals in the pedigree can be written as

(4) $$\left(\begin{array}{c} a_{1}\\ a_{2}\\ \cdots \end{array}\right)∼N\left(0,A⊗K_{g}\right)$$

In RR‐GBLUP, the pedigree‐based numerator relationship matrix **A** is replaced by a marker‐based genomic relationship matrix **G** to depict the genetic covariances between individuals.

### 2.2 RR‐MEM

**FIGURE 1:**
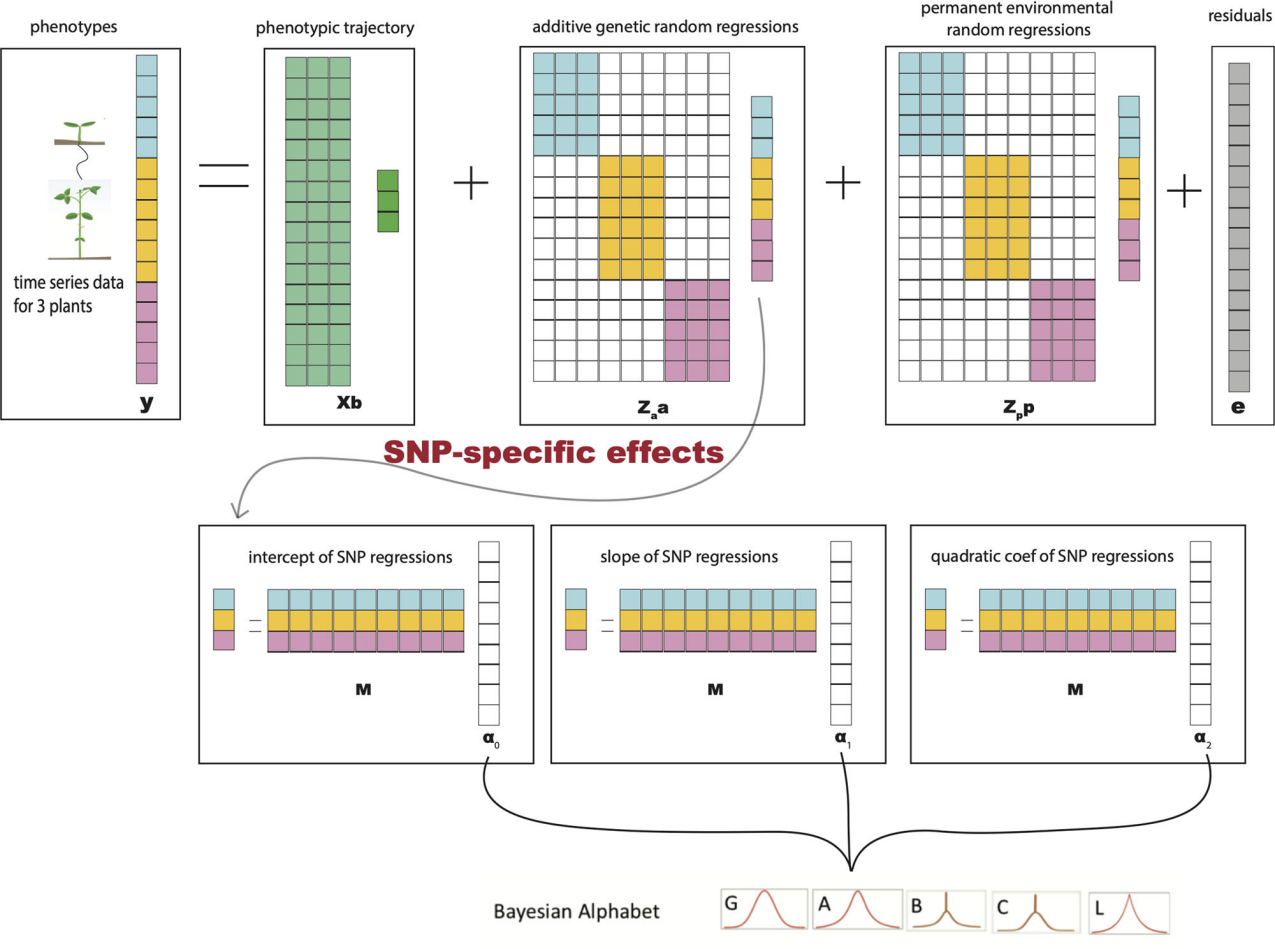
Graphic demonstration of the random regression marker effect model. In this example, quadratic regressions are assumed for fixed effects, additive genetic effects, and permanent environmental effects. Different prior distributions of marker effects can be incorporated in this model (e.g., normal distribution, BayesA prior, BayesB prior, BayesC prior, and Bayesian LASSO prior). SNP, single‐nucleotide polymorphism

In RR‐GBLUP, which incorporates the genomic relationship matrix in RRM, individuals' temporal additive genetic effects are captured by individual random regressions, with each individual having its own regression coefficients estimated. Here, we re‐parameterize the coefficients of additive genetic random regressions in RR‐GBLUP as multi‐regressions on marker covariates. That is, Equation 1 can be re‐parameterized as

(5) $$y_{ikt}=\sum_{l=0}^{m_{b}}b_{kl}x_{klt}+\sum_{l=0}^{m_{a}}z_{a_{ilt}}\sum_{j=1}^{p}m_{ij}\alpha_{lj}+\sum_{l=0}^{m_{p}}p_{il}z_{p_{ilt}}+e_{ikt}$$

where *p* is the total number of markers, *m_ij_* is the marker covariate at locus *j* for individual *i*, and α*_lj_* is the marker effect of locus *j* corresponding to the (*l* + 1)th random regression coefficients of additive genetic effects. Other parameters in Equation 5 stay the same as in Equation 1. In matrix notation, RR‐MEM for observations of individual *i* can be written as

(6) $$\begin{array}{c} y_{i}=X_{i(t)}b_{k}+Z_{a_{i}\left(t\right)}\sum_{j=1}^{p}m_{\mathrm{ij}}\alpha_{j}+Z_{p_{i}\left(t\right)}p_{i}+e_{i} \end{array}$$

where **α** *_j_* is a vector of length (*m*_a_ + 1) of random regression coefficients for locus *j*. Other parameters stay the same as in Equation 2. A graphic demonstration of RR‐MEM is shown in Figure 1.

We incorporate Bayesian variable selection methods into RRM by using the mixture priors described in Cheng, Kizilkaya, et al. (2018). We write the vector of marker effects corresponding to all additive genetic random regression coefficients at locus *j* as **α** *_j_* = **D** *_j_* **β** *_j_*, where **D** *_j_* is a diagonal matrix with *c*th diagonal element indicating whether the marker effect for the *c*th random regression coefficients is zero or not, and **β** *_j_* follows a multivariate normal distribution with null mean and covariance matrix **K**_α_ denoting the marker effects for locus *j* when it influences all the regression coefficients of additive genetic effects.

If quadratic regressions are used for the additive genetic effects, a total of 2^3^ = 8 possible outcomes can be observed for the diagonals of **D** *_j_*. For example, one of the possible outcomes for **D** *_j_* can be Diag(0,0,1), which indicates that locus *j* does not contribute to the intercept and slope of the additive genetic effects but only has effect on the quadratic coefficients of the additive genetic effects. At each iteration of MCMC chains, each outcome has its own probability (i.e., π) sampled across the genotyped loci. Based on the posterior probabilities of the outcomes, a general pattern of the functionality of markers toward additive genetic random regressions can be inferred. Note that if **D** *_j_* is predefined as an identity matrix for all markers (i.e., variable selection is not performed), RR‐MEM is equivalent to RR‐GBLUP in terms of prediction of breeding values.

Besides marker effects, in RR‐MEM, flat priors are used for the fixed regression coefficients; a multivariate normal distribution is used for the regression coefficients of permanent environmental effects; and heterogeneous variances are assumed for the residuals. In this paper, the mixture prior of multi‐trait BayesC is used for **α** *_j_* in RR‐MEM (hereafter referred to as RR‐BayesC), and its conditional posterior distributions used in Gibbs samplers are derived in the following section. It should be straightforward to implement other Bayesian priors for **α** *_j_* in a same manner.

### 2.3 Posterior distributions for Gibbs sampler

Let **δ** *_j_* be a vector containing the diagonal elements of **D** *_j_*. The conditional posterior distributions of **δ** *_j_* and **β** *_j_* in Gibbs samplers are given below, and their derivations in RR‐BayesC are provided in the Supplemental Material. Due to the high correlation between **β** *_j_* and **δ** *_j_*, **β** *_j_* and **δ** *_j_* are sampled jointly from their joint conditional posterior distribution (Cheng, Kizilkaya, et al., 2018). Let **θ** denote all other parameters except for **β** *_j_* and **δ** *_j_*; the joint conditional posterior distribution for **β** *_j_* and **δ** *_j_* can be expressed as:

(7) $$\begin{array}{c} f\left(\beta_{j},\delta_{j}|\theta ,y\right)=f\left(\delta_{j}|\theta ,y\right)f\left(\beta_{j}|\delta_{j},\theta ,y\right) \end{array}$$

The full conditional posterior distribution of **β** *_j_* is

(8) $$f\left(\beta_{j}|\delta_{j},\theta ,y\right)\propto N\left(C_{j}^{-1}r_{j},C_{j}^{-1}\right)$$

where $C_{j}=D_{j}^{\prime }\left[\sum_{i=1}^{n}Z_{a_{i}\left(t\right)}^{\prime }\left(R^{-1}\right)Z_{a_{i}\left(t\right)}m_{\mathrm{ij}}^{2}\right]D_{j}+K_{\alpha }^{-1}$, $r_{j}=D_{j}^{\prime }\left[\sum_{i=1}^{n}m_{\mathrm{ij}}Z_{a_{i}\left(t\right)}^{\prime }\left(R^{-1}\right)w_{\mathrm{ij}}\right]$, and $w_{\mathrm{ij}}=y_{i}-X_{i(t)}b_{k}-Z_{p_{i}\left(t\right)}p_{i}-\sum_{l\neq j}m_{\mathrm{il}}Z_{a_{i}\left(t\right)}D_{l}\beta_{l}$.

The marginal full conditional posterior distribution of **δ** *_j_* is

(9) $$\begin{array}{cc} f(\delta_{j}|\theta ,y) & =\frac{f\left(y|\theta ,\delta_{j}\right)f\left(\delta_{j}|\pi \right)}{{\Sigma_{\delta }}_{j}f\left(y|\theta ,\delta_{j}\right)f\left(\delta_{j}|\pi \right)} \end{array}$$

with

(10)

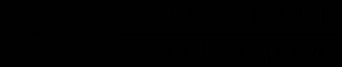

where *i* is a label that denotes the *i*th outcome from the $2^{m_{a}+1}$ possible outcomes of **δ** *_j_*.

### 2.4 Data analysis

In order to evaluate the performance of RR‐BayesC in genome‐enabled analysis, including both genomic prediction and association inference, a simulated study and real data analysis were conducted based on information from 357 accessions in Rice Diversity Panel 1 (RDP1) (Campbell et al., 2018; Zhao et al., 2011).

#### 2.4.1 Genomic prediction

**FIGURE 2:**
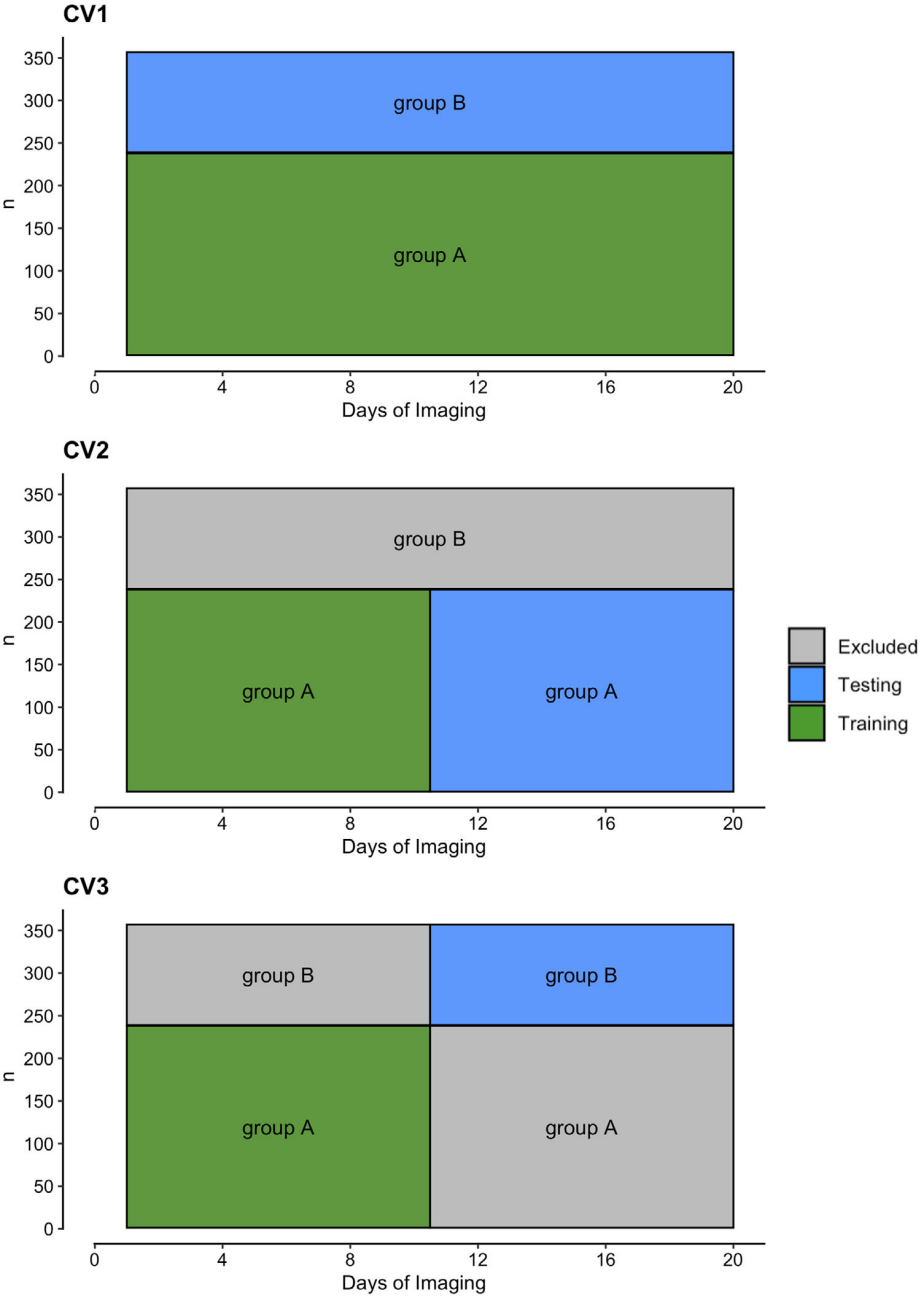
Graphic representation of cross‐validation schemes for genomic prediction of longitudinal traits. CV1: all phenotypic records of individuals in Group A were used to predict the performance of Group B, which have no observations available. CV2: earlier phenotypic records of individuals in Group A were used to forecast the future performance of same individuals. CV3: earlier phenotypic records of individuals in Group A were used to forecast the future performance of individuals in Group B

For genomic prediction, three cross‐validation (CV) schemes were used to evaluate the performance of RR‐BayesC (Figure 2). All 357 accessions were split into two groups. Group A consists of 238 accessions (two‐third of the data), and Group B consists of the remaining 119 accessions (one‐third of the data). In the first scheme (CV1), phenotypes at all 20 time points of Group A were used as training data, and phenotypes at all 20 time points of Group B were used for validation. This CV scheme mimics a scenario when all the observations of “old” accessions are used to predict the performance of “new” accessions that do not have any observations available. In the second scheme (CV2), only individuals in Group A were used for the CV procedure. Phenotypes at the first 10 time points of Group A were used as training data, and phenotypes at the last 10 time points from the same accessions were used for validation. This CV scheme mimics a simple forecasting scenario when future phenotypes of “old” accessions are not available and are predicted from their own earlier performance. In the third scheme (CV3), phenotypes at the first 10 time points of Group A were used as training data, and phenotypes measured at the last 10 time points of Group B were used for validation. This CV scheme mimics a different forecasting scenario when future phenotypes of “new” accessions are predicted from the earlier phenotypes of “old” accessions.

Markov chain Monte Carlo chains of length 50,000 were run for RR‐BayesC, with the first 10,000 iterations discarded as burn‐in. A thinning rate of 50 was used for computing posterior means as estimates of parameters. The random regression coefficients of additive genetic effect for individual *i* (**a** *_i_*) in a validation set were calculated as **a** *_i_* = **M** *_i_* **α**, where **M** *_i_* = **I**_3_ ⊗ **m** *_i_^T^* and **α** = [**α**_0_ *^T^* **α**_1_ *^T^* **α**_2_ *^T^*]*^T^*, with **m** *_i_* being the marker covariates of individual *i* and **α** *_l_* being the marker effects for the (*l* + 1)th random regression coefficients. Then, the breeding value of individual *i* at time *t* was computed as the dot product of $z_{a_{t}}$ and **a** *_i_*, where $z_{a_{t}}$ is a vector of Legendre polynomials corresponding to time *t*. Pearson correlation between estimated breeding values and observed phenotypes at each day was used as a measurement of predictive accuracy.

#### 2.4.2 Association inference

In Bayesian variable selection regression models, posterior inclusion probability (PIP) is usually used for the inference of single markers. The contribution of a genomic window may also be considered for association inference because SNPs within a genomic window may jointly capture the signals from causal variants (Fernando et al., 2017). Posterior probability of association of a genomic window (WPPA) (Fernando et al., 2017; Li et al., 2021) was proposed for tests of association based on genomic windows. In our study, both PIP and WPPA are used.

The performance of association inference to detect causal variants was compared between RR‐BayesC and single‐trait BayesC (ST‐BayesC). For ST‐BayesC, phenotypes observed at each time point were treated as an independent trait for single‐trait analysis. The model specification of ST‐BayesC is provided in the Supplemental Material. Markov chain Monte Carlo chains of length 100,000 were run for RR‐BayesC and ST‐BayesC, with the first 50,000 iterations discarded as burn‐in. A thinning rate of 100 was used for computing posterior means as estimates of parameters.

In order to compare the performance of association inference between RR‐BayesC and ST‐BayesC, time‐specific marker effects were calculated from SNP‐specific effects estimated from RR‐BayesC. The time‐specific effect of locus *j* at time *t* estimated from RR‐BayesC is computed as $\alpha_{tj}=\sum_{l=0}^{m_{a}}z_{a_{tl}}\alpha_{lj}$, where $z_{a_{tl}}$ is the *l*th order Legendre polynomial corresponding to time *t*, and α*_lj_* is the marker effect of locus *j* corresponding to the (*l* + 1)th random regression coefficients of additive genetic effects. Based on the estimated time‐specific marker effects, the PIP of single markers and the WPPA of genomic windows at each day were computed using the MCMC samples of marker effects generated from the Bayesian variable selection models (i.e., RR‐BayesC and ST‐BayesC). For RR‐BayesC, both PIP and WPPA based on time‐specific marker effects and PIP based on SNP‐specific marker effects were studied for association inference.

#### 2.4.3 Simulated study

In this section, datasets are simulated based on real genotypes of 33,690 SNPs from the 357 accessions in RDP1. Phenotypic trajectory was described by a second‐order polynomial function of time, with the fixed regression coefficients sampled from a univariate normal distribution with null mean and standard deviation equal to 10. In addition, quadratic random regressions of additive genetic effects were used to simulate the temporal genetic variation. Ten randomly selected quantitative trait loci (QTL) were simulated to jointly influence the additive genetic random regression coefficients, whose effects were sampled from a multivariate normal distribution with null mean and (co)variance matrix:

(11) $$K_{\alpha }=\left[\begin{array}{ccc} 1.0 & 0.5 & 0.2\\ 0.5 & 1.0 & 0.8\\ 0.2 & 0.8 & 1.0 \end{array}\right]$$

In addition to the 10 QTL that exert pleiotropic effects on the random regression coefficients, 10 other QTL were simulated to specifically influence each of the random regression coefficients (i.e., intercept, slope, and quadratic regression coefficient). Phenotypes at 20 time points (days) were simulated with consistent residuals across time points. To simulate different heritability across time points, heritability at Day 10 was set as *h* ^2^_10_ = 0.5, and the same residual variance was set to be σ^2^ *_e_* = [(1 − *h* ^2^_10_)/*h* ^2^_10_]σ^2^ *_g_*_,10_ for all 20 time points, with σ^2^ *_g_*_,10_ being the genetic variance at Day 10. A detailed simulation of individual temporal additive genetic effects is provided in the Supplemental Material. In this simulated study, 20 replicated datasets were simulated. For the model specification of RR‐BayesC in this simulated study, Legendre quadratic regressions were fitted to both fixed effects and additive genetic effects. Consistent residual variances were assumed across the 20 time points.

In genomic prediction, for each of the 20 simulated populations, five folds were run for each CV scheme. Therefore, 100 runs were conducted for each CV scheme in our simulated study. In CV1, the performance of RR‐BayesC is compared with the performance of single‐trait models (including single‐trait BayesC and single‐trait GBLUP). In CV2, the performance of RR‐BayesC is compared with that of the repeatability model. Average prediction accuracy over the 100 runs was used to compare the performance of different models. Model specifications of single‐trait GBLUP (ST‐GBLUP), single‐trait BayesC (ST‐BayesC), and repeatability models are provided in Supplemental Material.

In the association analysis, all QTL that affect the random regression coefficients of additive genetic effects were considered as true causal variants for the simulated longitudinal trait. Non‐overlapping genomic windows containing 100 SNPs were tested, and those explaining more than 0.3% (∼1/*n*_w_, with *n*_w_ being the number of genomic windows) of total genetic variance were considered to be potential genomic windows that contain causal variants.

The relative performance of association inference of ST‐BayesC and RR‐BayesC was based on partial area under the receiver operating characteristic curve (pAUC). Compared with area under the entire receiver operating characteristic curve, pAUC focuses more on the regions with relatively high levels of specificity (Ma et al., 2013). Specifically, a maximum false‐positive rate of 5% was used as the cutoff to compute the pAUC (i.e., pAUC05) in our simulated study. For the ease of interpretation, the computed pAUC05 values in our simulated study were rescaled such that pAUC05 of a random classifier is equal to 1 (Chen et al., 2017).

#### 2.4.4 Real data analysis

Real phenotypes and genotypes from the 357 accessions of RDP1 (Zhao et al., 2011) were analyzed using the phenotypic data provided in Baba et al. (2020). Three experiments with partially replicated design were conducted at the Plant Accelerator Australian Plant Phenomics Facility in University of Adelaide, SA, Australia, from February to April 2016. Two smart houses were used to grow the rice accessions. A detailed description of the experimental design is provided in Baba et al. (2020).

Shoot biomass, which is measured by projected shoot area (PSA) through image‐based phenotyping, and water usage (WU), which is collected through automated watering system, were used as target longitudinal traits in our real data analysis. In each experiment, plants were imaged daily over a period of 20 d (from 13 to 33 d after transplant) using red‐green‐blue cameras. Two side‐view images and a top‐view image were collected daily for each plant, and its daily PSA was computed as the sum of plant pixels extracted from the images and used as its biomass phenotypic value. After imaging, water was added to each plant to reach a predefined weight, and an estimation of the amount of water that lost through evapotranspiration was computed for each day.

The PSA and WU measurements of accessions grown in the control condition were analyzed (Baba et al., 2020). Adjusted genotypic mean (i.e., BLUE) of each accession and at each day was used as our response variable. The BLUE values were obtained using Equation 12.

(12) $$P_{itklr}=\mu +A_{i}+D_{t}+AD_{it}+E_{k}+AE_{ik}+S_{l}+e_{itklr}$$

where *P_itklr_* is the PSA/WU value of the *r*th replicate of accession *i* observed at day *t* that was planted in experiment *k* in smart house *l*, μ is the overall mean, *A_i_* is the individual effect of accession *i*, *D_t_* is the effect of day *t*, *AD_it_* is the interaction between accession *i* and day *t*, *E_k_* is the effect of experiment *k*, *AE_ik_* is the interaction between accession *i* and experiment *k*, *S_l_* is the effect of smart house *l*, and *e_itklr_* is the residual effect. Among these parameters, μ, *A_i_*, *D_t_*, and *AD_it_* are treated as fixed effects, and *E_k_*, *AE_ik_*, and *S_l_* are treated as random effects. The adjusted genotypic mean *y_it_* for accession *i* at day *t* is computed as *y_it_* = μ + *A_i_* + *D_t_* + *AD_it_*.

After the adjustment that removed experimental effects, quadratic Legendre polynomials were used in RR‐BayesC. The RR‐BayesC model is written as

(13) $$y_{it}=\sum_{l=0}^{2}b_{l}x_{ilt}+\sum_{l=0}^{2}z_{a_{ilt}}\sum_{j=1}^{p}m_{ij}\alpha_{lj}+\sum_{l=0}^{2}p_{il}z_{p_{ilt}}+e_{it}$$

where *y_it_* is the BLUE of PSA or WU for accession *i* at day *t*; $x_{ilt}$, $z_{a_{ilt}}$, and $z_{p_{ilt}}$ represent the *l*th order Legendre polynomial covariate corresponding to day *t* for the fixed regression, additive genetic random regression, and permanent environmental random regression of accession *i*, respectively; *b_l_* is the (*l* + 1)th fixed regression coefficient that accounts for the overall mean; *m_ij_* is the marker covariate at locus *j* for individual *i*; α*_lj_* is the marker effect of locus *j* corresponding to the (*l* + 1)th random regression coefficients of additive genetic effects; *p_il_* is the (*l* + 1)th random regression coefficient of permanent environmental effect for individual *i*; and *e_it_* is the residual. Heterogeneous residual variance is assumed across the 20 time points. The random regression coefficients of permanent environmental effects are treated as independent variables. Based on the model described in Equation 13, the performance of RR‐BayesC for association inference and genomic prediction was studied. For genomic prediction, 20 replicates were conducted for each CV scheme. In CV1, the performance of RR‐BayesC was compared with that of ST‐BayesC. In CV2, the performance of RR‐BayesC was compared with that of a repeatability model. The average prediction accuracy over the 20 replicates was used to compare the performance of different models. For association analysis, non‐overlapping genomic windows containing 100 SNPs were tested, and those that explained more than 1% of total genetic variance were considered to be potential genomic windows that contain QTL.

## 3 RESULTS

### 3.1 Simulated study

#### 3.1.1 Genomic prediction

**FIGURE 3:**
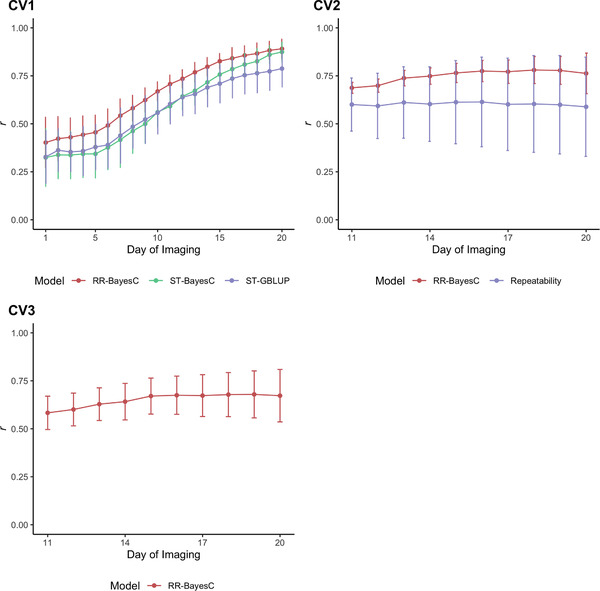
Genomic prediction accuracy under three different cross validation (CV) schemes for simulated study. CV1: phenotypic records for individuals in Group A were used to predict the performance of Group B, which have no observations available. The performance of three different models (i.e., RR‐BayesC, ST‐BayesC, and ST‐GBLUP) was compared. CV2: earlier phenotypic records of individuals in Group A were used to forecast the future performance of same individuals. The performance of RR‐BayesC and repeatability model were compared. CV3: earlier phenotypic records of individuals in Group A were used to forecast the future performance of individuals in Group B. Individuals were not overlapped between Group A and Group B

The prediction accuracies obtained for the simulated study under the three cross‐validation schemes (see Figure 2) are presented in Figure 3. In CV1, full records of 238 individuals (training individuals) were used to predict the performance of the remaining 119 individuals (testing individuals). For RR‐BayesC, all the records across 20 time points from training individuals were used to estimate the phenotypic trajectories of testing individuals. For ST‐BayesC and ST‐GBLUP, phenotypes of training individuals at a single day were used to predict the phenotypes of testing individuals at the same day. Overall, RR‐BayesC showed significantly higher prediction accuracy than the two single‐trait methods. The prediction accuracy for RR‐BayesC ranged from 0.40 to 0.89, whereas the prediction accuracy for ST‐BayesC and ST‐GBLUP ranged from 0.32 to 0.87 and from 0.33 to 0.79, respectively.

In CV2, future performance of known individuals was predicted from each individual's earlier performance. Phenotypes during the first 10 d of 238 individuals were used to estimate their own genetic trajectories by RR‐BayesC. Later, phenotypes of the same individuals were forecast based on their estimated random regressions of additive genetic effects. The prediction accuracy for RR‐BayesC increased from 0.69 to 0.78 as the number of days increased, which indicates that accurate estimation of genetic trajectories was achieved for the 238 individuals. On the other hand, although *h* ^2^ increases as time progresses, the prediction accuracy of the repeatability model slightly decreased from 0.60 at Day 11 to 0.59 at Day 20. Due to the relatively low *h* ^2^ for phenotypes at the earlier period, which is used as a training dataset, the standard errors of the prediction accuracy at later periods by the repeatability model are relatively large.

In CV3, the future performance of individuals without any phenotypic records was predicted using earlier phenotypes of correlated individuals. Phenotypes during the first 10 d of 238 individuals were used to predict the genetic trajectories of the remaining 119 individuals that have no phenotypes observed. Note that the standard single‐trait or repeatability models are not applicable in this scenario because observations at later time points are not available for training individuals and no observations are available for testing individuals. Through RR‐BayesC, the random regressions of additive genetic effects for testing individuals were estimated, leading to prediction accuracies ranging from 0.58 to 0.68.

#### 3.1.2 Association inference

For RR‐BayesC, Legendre quadratic regressions were fitted to both fixed effects and additive genetic effects. Consistent residual variances were assumed across the 20 time points.

To compare the performance of association inference between RR‐BayesC and ST‐BayesC, time‐specific marker effects for locus j were calculated from its SNP‐specific effects estimated from RR‐BayesC as $\sum_{l=0}^{2}z_{a_{tl}}\alpha_{lj}$. The PIP of single markers and the WPPA of genomic windows at each day were used to compare RR‐BayesC and ST‐BayesC. Average rescaled pAUC05 values over 20 replicates were used to measure the general performance of RR‐BayesC and ST‐BayesC.

**FIGURE 4:**
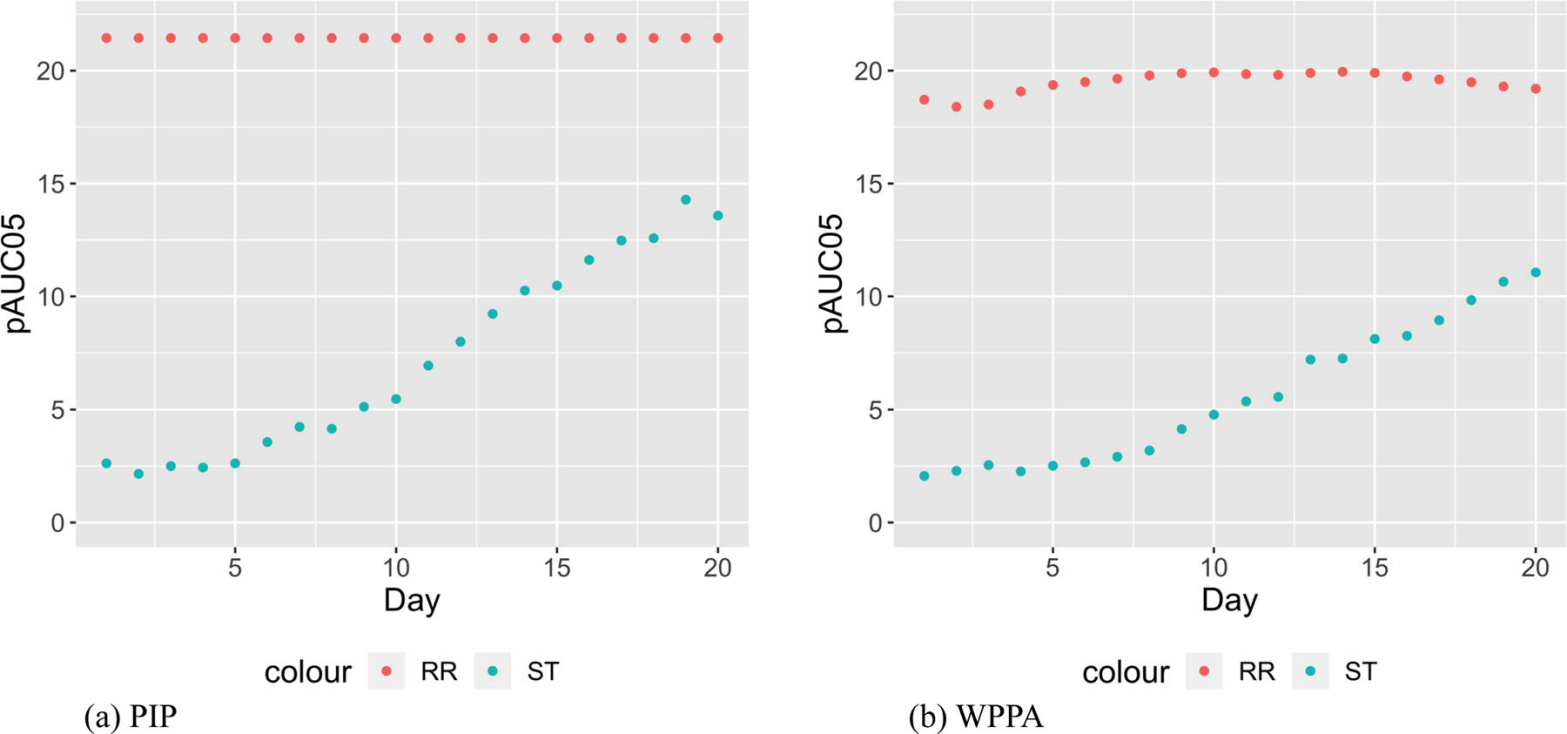
Comparison of association inference between RR‐BayesC and ST‐BayesC using average rescaled pAUC05 based on (a) posterior inclusion probability (PIP) and (b) window posterior probability of association (WPPA), respectively

Figure 4 shows the average rescaled pAUC05 of ST‐BayesC and RR‐BayesC based on PIP and WPPA, respectively. Generally, better association performance has been demonstrated for RR‐BayesC using either PIP or WPPA. For ST‐BayesC, average rescaled pAUC05 increased from Day 1 to Day 20 for both PIP and WPPA as *h* ^2^ increased. The average rescaled pAUC05 of ST‐BayesC ranged from 2.15 to 14.29 and from 2.06 to 11.06 when PIP and WPPA was used for inference, respectively. For RR‐BayesC, average rescaled pAUC05 values based on PIP were consistent at 21.45 across the 20 d. Because the Legendre polynomials (i.e., $z_{a_{tl}}$) are non‐zero values, the time‐specific PIP of SNP *j* is determined by its SNP‐specific PIP, which is time invariant. However, time‐specific effects of SNPs are also used to calculate WPPA. The average rescaled pAUC05 based on WPPA for RR‐BayesC ranged from 18.39 to 19.92.

**FIGURE 5:**
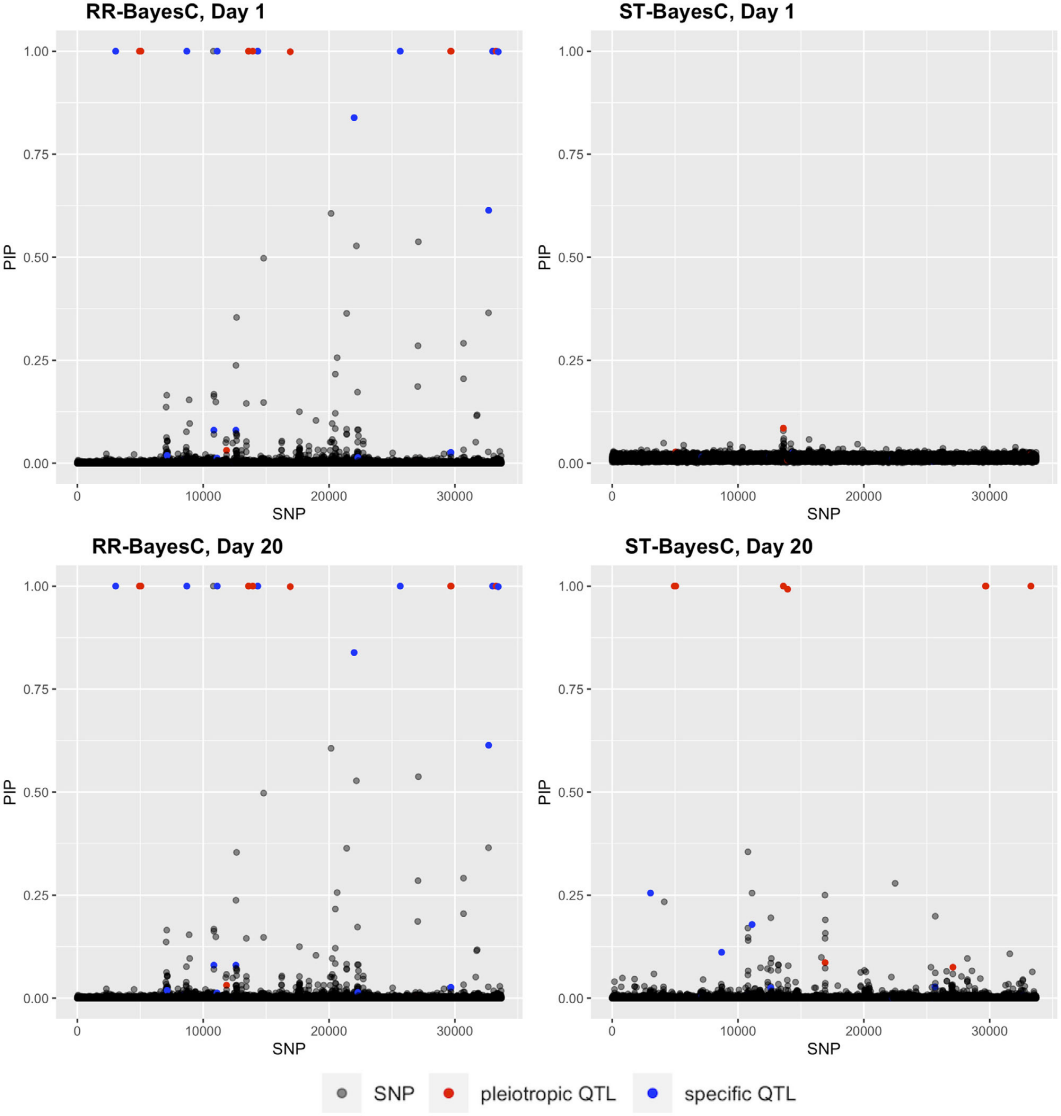
Posterior inclusion probability (PIP) of markers at Day 1 (first row) and Day 20 (second row) in simulated study. The performance of RR‐BayesC is shown in the left panel, and the performance of ST‐BayesC is shown in the right panel. Red dots specify the pleiotropic quantitative trait loci (QTL) that affect all the random regression coefficients of additive genetic effects, and blue dots specify the specific QTL that only affect one of the random regression coefficients of additive genetic effects

**FIGURE 6:**
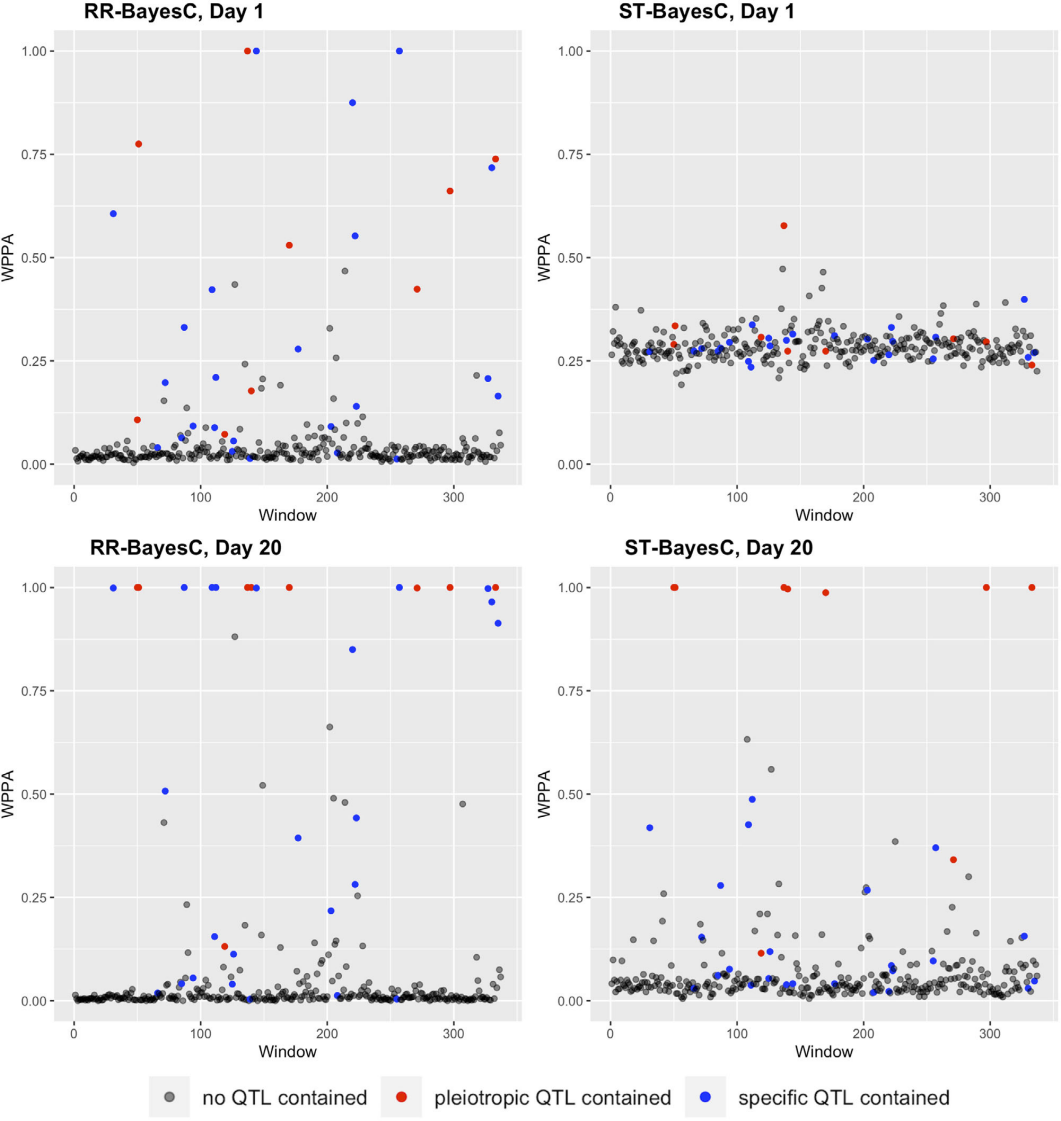
Window posterior probability of association (WPPA) of genomic windows at Day 1 (first row) and Day 20 (second row) in a simulated study. The performance of RR‐BayesC is shown at the left panel, and the performance of ST‐BayesC is shown at the right panel. Red dots specify the causal windows that contain pleiotropic quantitative trait loci (QTL), which affect all the regression coefficients of additive genetic effects. Blue dots specify the causal windows that contain specific QTL, which only affect one of the regression coefficients of additive genetic effects

A detailed performance of association inference for one of the replicated populations is shown in Figures 5 and  6. In our simulated study, *h* ^2^ was simulated to increase across time, with the highest *h* ^2^ achieved at Day 20. The PIPs of markers obtained by RR‐BayesC and ST‐BayesC at Days 1 and 20 are shown in Figure 5. In Figure 5, two types of QTL are highlighted with different colors: red is used to denote QTL that simultaneously affect all the regression coefficients of additive genetic effects, and blue is used for QTL that specifically affect one of the regression coefficients of additive genetic effects. Generally, ST‐BayesC is able to detect some of the pleiotropic QTL when *h* ^2^ is relatively high (i.e., at Day 20), whereas RR‐BayesC detects both pleiotropic QTL and the specific QTL that affect one of the regression coefficients of additive genetic effects. When PIP = 0.95 was used as the threshold for association inference, 17 SNPs were inferred as causal variants by RR‐BayesC, among which 15 were true positives. Seven of the 15 true positives were specific QTL that only affect one of the regression coefficients of additive genetic effects, and 8 of the 15 true positives were pleiotropic QTL that affect all the regression coefficients of additive genetic effects. For ST‐BayesC, no SNPs have passed the PIP threshold at Day 1. The performance of ST‐BayesC improved at Day 20, and seven pleiotropic QTL were correctly inferred.

In addition to the association inference of a single marker based on PIP, a window‐based association inference was shown for one of the replicated populations. The WPPAs of genomic windows obtained from RR‐BayesC and ST‐BayesC at Day 1 and Day 20 are shown in Figure 6. Unlike PIP, WPPAs of genomic windows in RR‐BayesC are affected by temporal covariates, and the contribution of genomic windows toward genetic variation changes across time. Windows containing QTL were divided into two groups in Figure 6, where windows containing pleiotropic QTL are designated by red color and windows containing specific QTL are designated by blue color. If a window contained both pleiotropic QTL and specific QTL, it was grouped as windows containing pleiotropic QTL and was designated by red color. Generally, the performance of ST‐BayesC and RR‐BayesC was improved as *h* ^2^ increased (i.e., at Day 20). Similar to the pattern observed in Figure 5, ST‐BayesC can mainly detect windows that contain pleiotropic QTL, whereas RR‐BayesC was able to detect windows that contain either pleiotropic QTL or specific QTL. A WPPA of 0.95 was used as the threshold for association inference. At Day 1, no significant windows were inferred by ST‐BayesC, and three genomic windows had their WPPA >0.95 in RR‐BayesC. Among the three significant windows inferred by RR‐BayesC, one contained pleiotropic QTL, and the others contained specific QTL. At Day 20, significant windows inferred by ST‐BayesC and RR‐BayesC were all true positives. For ST‐BayesC, seven windows that contain pleiotropic QTL were correctly inferred. For RR‐BayesC, among the 16 significant windows, eight contained pleiotropic QTL and eight contained specific QTL.

According to the GWAS performance in a simulated study, RR‐BayesC showed significant improvement in capturing the dynamic signal from causal variants. Besides the ability to identify more causal variants that influence temporal additive genetic effects, RR‐BayesC can distinguish causal variants that have interaction with time. That is, markers that only affect the intercept of additive genetic random regressions have constant effects on the longitudinal trait across time, whereas markers affecting the second or greater random regression coefficients have dynamic effects across time, presenting interaction with time. Furthermore, time‐specific marker effects at two distant time points differ dramatically for markers that affect higher‐order polynomial coefficients compared with markers that only affect lower‐order polynomial coefficients, even with same SNP‐specific effect size. Therefore, the identification of markers that affect different random regression coefficients of additive genetic effects plays an important role in the analysis of interaction between genetics and time, and the markers that affect higher‐order polynomial coefficients warrant further investigation.

**FIGURE 7:**
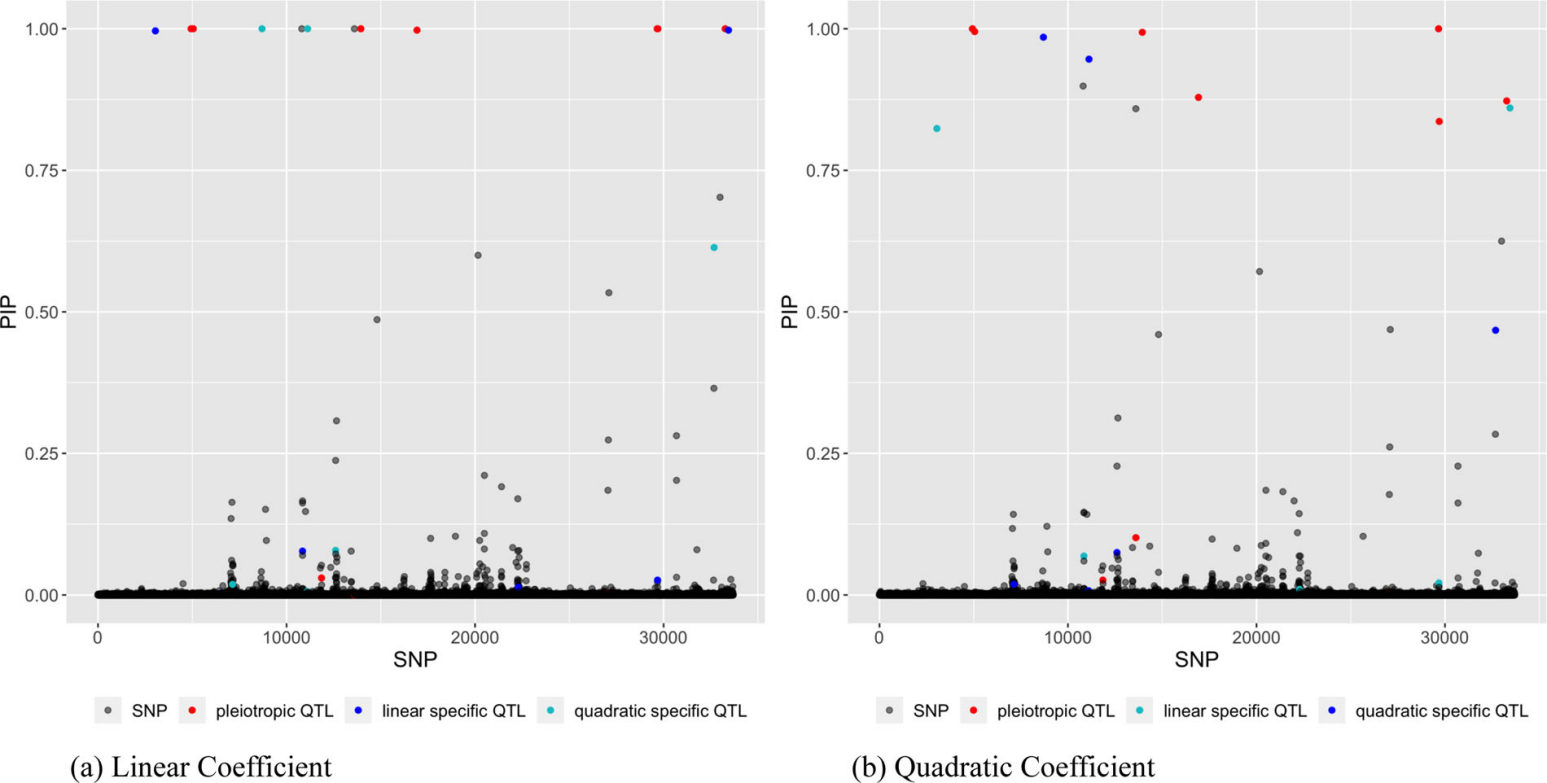
Posterior inclusion probability (PIP) of markers that affect random regression linear coefficients and random regression quadratic coefficients of additive genetic effects in simulated study. Red dots specify the pleiotropic quantitative trait loci (QTL) that affect all the regression coefficients of additive genetic effects, blue dots specify the specific QTL that only affect the corresponding regression coefficients of additive genetic effects, and turquoise dots specify the specific QTL that only affect the other regression coefficients of additive genetic effects. SNP, single‐nucleotide polymorphism

Figure 7a and Figure 7b show the PIP of markers that affect the linear and quadratic coefficients of additive genetic random regressions, respectively, for one of the replicated populations. For markers that affect the linear regression coefficients of additive genetic effects (Figure 7a), 13 significant associations were inferred, with a PIP threshold equal to 0.95. Nine out of the 13 significant associations are true positives, including the pleitropic QTL, which affect all the regression coefficients, and the specific QTL, which only affect the linear regression coefficients of additive genetic effects. Among the four false positives, two are specific QTL of the quadratic regression coefficients of additive genetic effects, and two are nonfunctional SNPs. For markers that affect the quadratic regression coefficients of additive genetic effects (Figure 7b), five significant associations were inferred, with a PIP threshold equal to 0.95, and all of them are true positives. Even though RR‐BayesC confused some specific QTL of additive genetic quadratic coefficients in the inference of QTL for linear coefficients, RR‐BayesC still, to an acceptable extent, demonstrated its potential to distinguish markers that have effects on different regression coefficients of additive genetic effects, especially the markers that affect higher order polynomial coefficients (i.e., quadratic coefficients).

### 3.2 Real data analysis

#### 3.2.1 Genomic prediction

**FIGURE 8:**
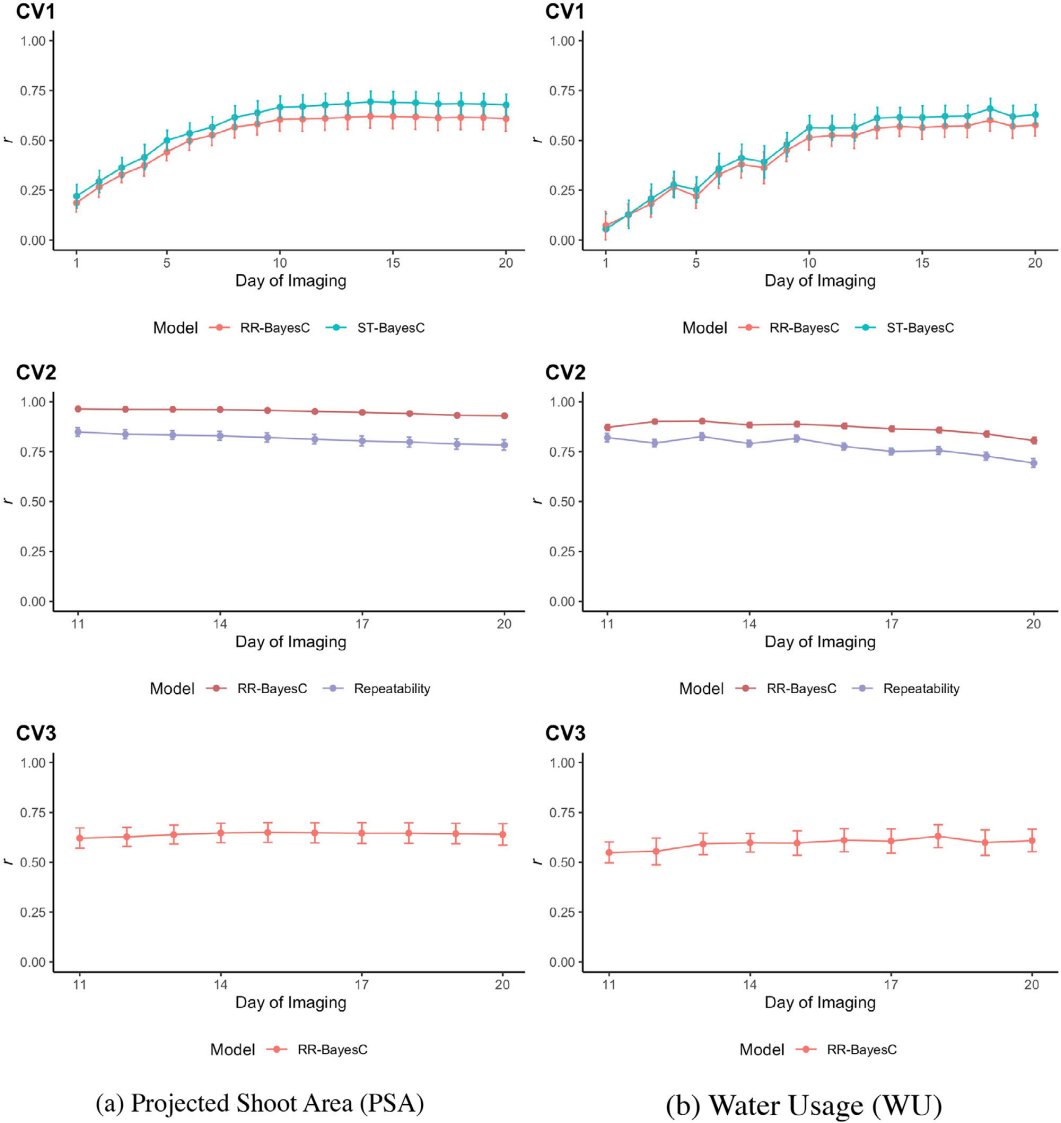
Genomic prediction accuracy under three different cross validation (CV) schemes for real data analysis of projected shoot area and water usage, respectively. CV1: full phenotypic records of individuals in Group A are used to predict the performance of individuals in Group B, who have no observations available. CV2: earlier phenotypic records of individuals in Group A were used to forecast their own future performance. CV3: earlier phenotypic records of individuals in Group A were used to forecast the future performance of individuals in Group B

The performance of RR‐BayesC for genomic prediction was demonstrated by real data analysis (Figure 8). When RR‐BayesC was used to predict the phenotypic trajectories of testing individuals using full records of training individuals (CV1), the prediction accuracy ranged from 0.19 to 0.62 and from 0.07 to 0.60 for PSA and WU, respectively. For both traits, the prediction accuracy increased significantly at an earlier period (i.e., from Day 1 to Day 10) and then maintained a relatively high value during the later period (i.e., from Day 11 to Day 20). For PSA, its prediction accuracy increased from 0.19 at Day 1 to 0.58 at Day 9 and then fluctuated at 0.61 (±0.01) from Day 10 to Day 20. For WU, its prediction accuracy increased from 0.07 at Day 1 to 0.51 at Day 10 and then ranged from 0.52 to 0.60 from Day 11 to Day 20. The performance of genomic prediction of RR‐BayesC was also compared with ST‐BayesC, whose prediction accuracy ranged from 0.22 to 0.69 and from 0.06 to 0.66 for PSA and WU, respectively.

In CV2, RR‐BayesC and the repeatability model were used to forecast the future performance of same individuals. Phenotypes measured during the first 10 d were used to predict the performance of same individuals during the last 10 d. Generally, RR‐BayesC outperformed the repeatability model through the later period. For PSA, the prediction accuracy during the last 10 d decreased from 0.98 to 0.94 and from 0.85 to 0.78 for RR‐BayesC and the repeatability model, respectively. A slight decline in predictive ability was observed for both methods as the days of imaging increased. This reduction is expected because the *h* ^2^ of PSA was consistent at the later period, and the phenotypic correlation between time points from training (Day 1 to Day 10) and testing (Day 11 to Day 20) datasets decreased as their distance increased. For WU, the prediction accuracy of RR‐BayesC increased from 0.87 at Day 11 to 0.90 at Day 13 and then decreased to 0.81 at Day 20, and the prediction accuracy of the repeatability model ranged from 0.69 to 0.83 at the later period.

In CV3, future performance of individuals without any observations was predicted by RR‐BayesC using earlier phenotypic records of other related individuals. The phenotypes of training individuals measured during the first 10 d were used to predict the performance of testing individuals during the last 10 d. The prediction accuracy for the last 10 d ranged from 0.65 to 0.67 and from 0.54 to 0.63 for PSA and WU, respectively.

#### 3.2.2 Association inference

**FIGURE 9:**
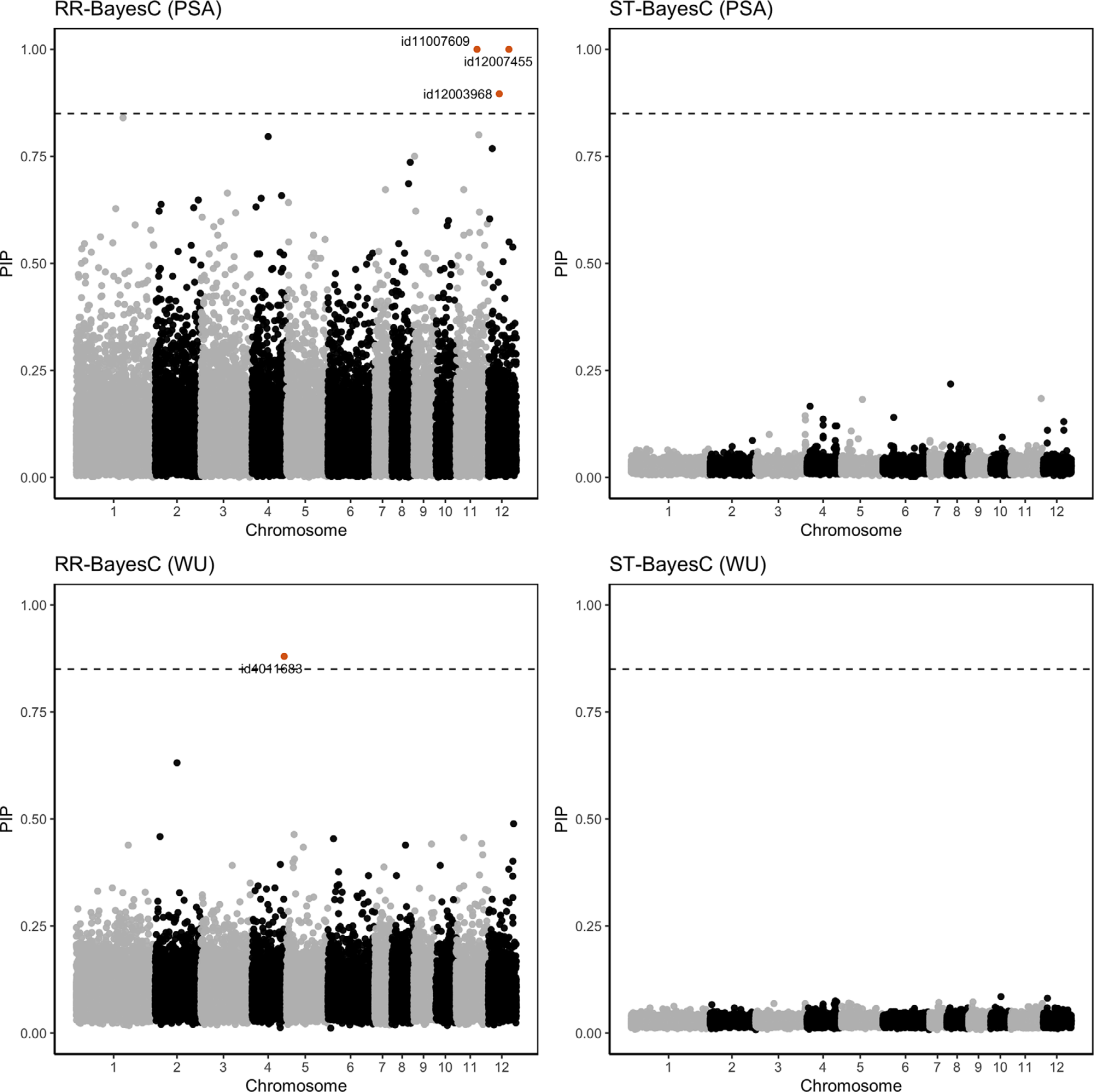
Posterior inclusion probability (PIP) of markers at Day 20 for projected shoot area (PSA) (first row) and water usage (WU) (second row) in real data analysis. The performance of RR‐BayesC is shown at the left hand side and the performance of ST‐BayesC is shown at the right hand side. Single‐nucleotide polymorphisms (SNPs) with PIP >0.85 are highlighted

In this section, genome‐wide association inference was conducted for real rice data. Figure 9 shows the PIP of time‐specific marker effects at Day 20 for PSA and WU. The performance of RR‐BayesC and ST‐BayesC was compared. A PIP at 0.85 was used as a threshold for association inference. In ST‐BayesC, no significant SNPs were inferred for either PSA or WU. In RR‐BayesC, three significant SNPs were inferred for PSA, and one significant SNP was inferred for WU. For PSA, the three significant SNPs were id11007609 on Chromosome 11 and id12003968 and id12007455 on Chromosome 12. For WU, the only significant SNP was id4011683 at Chromosome 4. Generally, based on time‐specific PIP, RR‐BayesC shows greater power to detect potential signals compared with ST‐BayesC.

For window‐based association, genomic windows that explained more than 1% of total genetic variance were considered to be potential genomic windows that contain causal variants. However, no significant windows were detected based on their time‐specific WPPA for either PSA or WU (Supplemental Figure S1).

**FIGURE 10:**
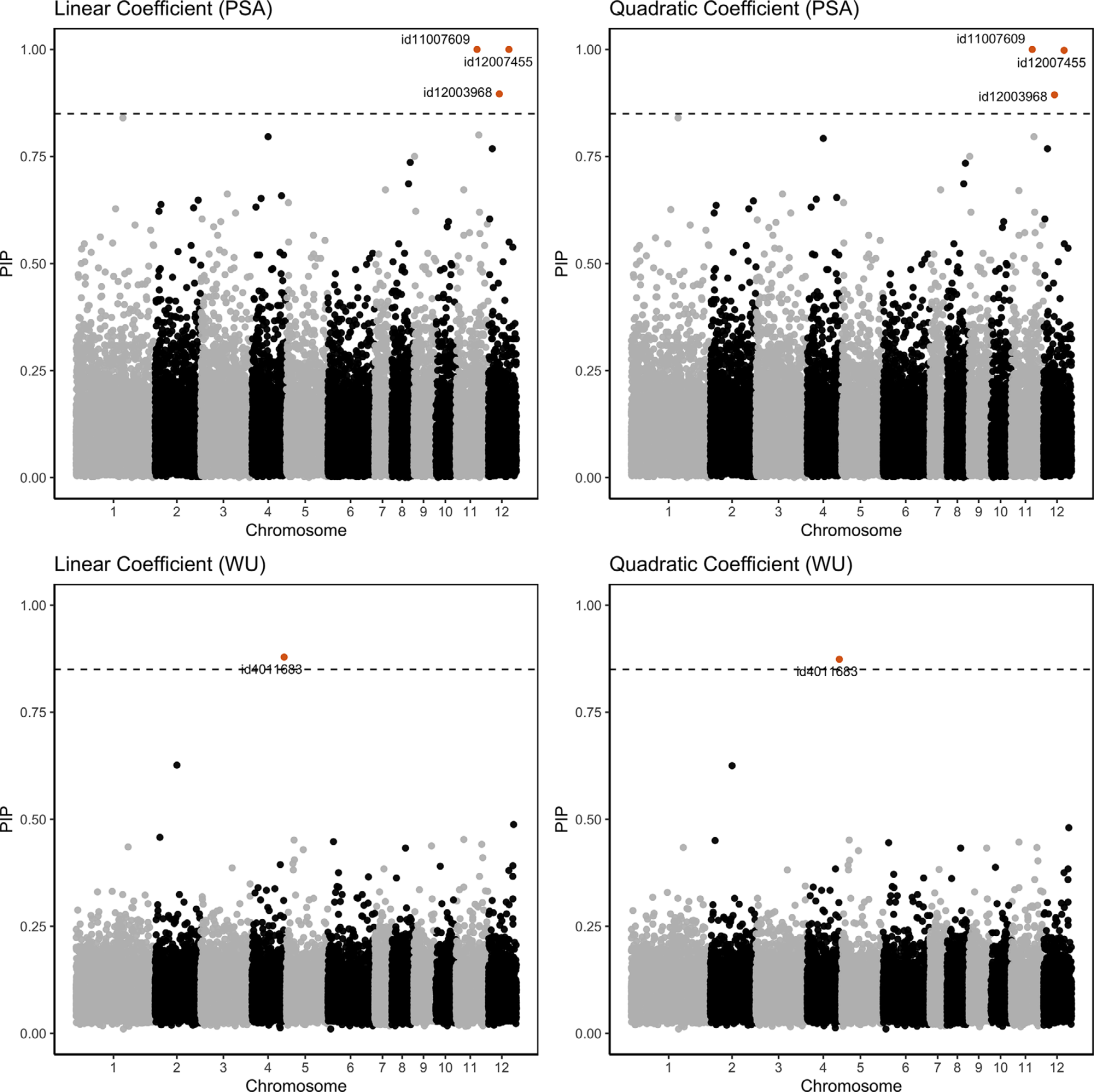
Posterior inclusion probability (PIP) of markers that affect random regression linear coefficients and random regression quadratic coefficients of additive genetic effects for projected shoot area (PSA) (first row) and water usage (WU) (second row), respectively, in real data analysis. Single‐nucleotide polymorphisms (SNPs) with PIP >0.85 are highlighted

The PIPs of SNP‐specific marker effects that affect the linear and quadratic coefficients of additive genetic random regressions are shown in Figure 10. Similar PIP patterns are observed for both linear and quadratic regression coefficients of additive genetic effects. This results indicate that the QTL of PSA or WU jointly influence the regression coefficients of their genetic trajectories. In addition, extremely high correlations between the regression coefficients of additive genetic effects are observed for both PSA and WU (see Supplemental Material), which validates the high dependence between their regression coefficients shown in Figure 10.

## 4 DISCUSSION

Currently, most studies about temporal SNP‐specific effects are conducted under a frequentist framework. In addition to the study of temporal SNP‐specific effects under a frequentist‐based RRM (Ning et al., 2017; Zhang et al., 2021), environmental SNP‐specific effects have been studied under the frequentist‐based reaction norm model in the past few years (Streit et al., 2013). The reaction norm model that is generally used in G × E interaction analyses can be deemed as an RRM, where linear random regressions of environmental covariates are used to account for the variation of additive genetic effects under different environments (Schaeffer, 2004). Built upon RRM, the Bayesian variable selection model proposed in this paper (i.e., RR‐BayesC) allows the estimation of SNP‐specific effects based on mixture priors, which enables the identification of SNPs that interact with time and the estimation of breeding values. Besides temporal covariates, environmental covariates can be similarly fitted in RR‐BayesC for the genome‐enabled analysis of environmental data.

The performance of genomic prediction using RR‐BayesC was studied through simulated and real data analysis. In the simulated study, when the phenotypic and genetic trajectories were correctly modeled, RR‐BayesC outperformed ST‐BayesC in CV1. In CV2 and CV3, even though the *h* ^2^ during the earlier training period was relatively low, the prediction accuracy increased during the later period, showing that RR‐BayesC is able to accurately capture the genetic trajectories of individuals. In the real data analysis, the performance of ST‐BayesC outperformed RR‐BayesC in CV1. Compared with ST‐BayesC, a stronger modeling assumption is used in RR‐BayesC, assuming that the genetic and environmental regression functions we used to depict the temporal observations are close to the underlying biological mechanisms, whereas ST‐BayesC does not assume any dynamic architecture for the longitudinal trait. Therefore, it is understandable that if the regression functions we used do not closely match the underlying biological mechanisms, ST‐BayesC may outperform RR‐BayesC. Furthermore, it also indicates that different regression functions can give varied prediction accuracies. For example, the performance of RR‐BayesC under CV1 for PSA using different B‐spline functions (De Boor & De Boor, 1978) as the genetic and environmental regression functions are shown in Supplemental Figure S2. Corresponding deviance information criterion values (Supplemental Table S1) were computed for the comparison of RR‐BayesC models with different regression functions. Lower deviance information criterion values were observed for fourth‐order regression functions (i.e., linear B‐spline function with two knots and quadratic B‐spline functions with one knot). Estimates of heterogeneous residual variances with different regression functions are also included in Supplemental Table S1.

One major benefit of RRM is to predict the individual genetic merit at future period when there are no or few phenotypes recorded. The prediction accuracy of RR‐BayesC for testing individuals over the last 10 d was similar when either the full records of training individuals (CV1) or only earlier records of training individuals (CV3) were used to train the model. This indicates that RR‐BayesC is able to predict future phenotypes of “new” individuals with reasonably high accuracy by using only the earlier observations from “old” individuals.

A genome‐wide prediction of PSA based on frequentist‐based RRM was conducted by Campbell et al. (2018), which shows similar predictive performance as RR‐BayesC during the later period. Specifically, in CV1, although RRM achieved higher prediction accuracy than RR‐BayesC for the earlier period, the prediction accuracy between RRM and RR‐BayesC became approximately equivalent during the later period. In CV2, RR‐BayesC achieved higher prediction accuracy than RRM. In CV3, a similar prediction accuracy achieved by RR‐BayesC and RRM. In general, RR‐BayesC tends to show better predictive performance during the later period, when the interaction between time and SNPs is expected to be relatively large compared with the interaction during the earlier period. Although similar predictive performance was observed for RRM and RR‐BayesC, RR‐BayesC presents a potential impact on the identification of a QTL × time interaction.

The performance of genetic inference of RR‐BayesC was also studied through simulated study and real data analysis. In the simulated study, RR‐BayesC showed significant improvement in capturing true genetic associations. It has been shown that considering temporal covariates in GWAS can improve the ability to recover genetic associations for longitudinal traits (Campbell et al., 2019; Das et al., 2011). Compared with ST‐BayesC, the use of all records across time points in RR‐BayesC allows the recovery of genetic associations, especially at the earlier period when *h* ^2^ is too low for ST‐BayesC to detect any significant SNPs. In addition to time‐specific SNP effects, SNP‐specific effects are estimated by RR‐BayesC, which enables the identification of SNPs that interact with time. Moreover, SNP‐specific effects that correspond to higher‐order polynomial coefficients tend to play a more important role in the interaction analysis of genetics and time. In real data analysis, significant SNPs with high PIP values are inferred by RR‐BayesC, whereas no significant SNPs are detected by ST‐BayesC. These results are consistent with results from the simulated study, which indicate a better recovery of genetic associations in RR‐BayesC.

An important goal in plant breeding is to improve and forecast the performance of longitudinal traits (e.g., biomass production for vegetative plants and yield production for grain crops). Traditionally, these traits are genetically evaluated based on their accumulated production at a specific time point (e.g., the end of a cropping season) or using repeatability models treating observations at different time points as repeated measurements. However, the genetic variation of these dynamic traits can be attributed to the collective response of different genes that function at different periods of a physiological cycle. Although conventional RRM allows the estimation of temporal breeding values for the longitudinal traits, it cannot implement different biologically motivated assumptions to the underlying genetic structures and analyze the interaction between genes and time. On the contrary, the Bayesian random regression model (i.e., RR‐MEM) proposed in this paper not only enables the estimation of breeding values but also serves as a scaffold for eliciting Bayesian priors on the underlying causal variants and analyzing the interactions with time. In addition to the mixture prior from BayesC, RR‐MEM is able to implement a collection of prior distributions in random regression models.

The software tool JWAS offers routines to perform such random regression analyses.

## AUTHOR CONTRIBUTIONS

Jiayi Qu: Conceptualization; Formal analysis; Methodology; Software; Validation; Visualization; Writing – original draft. Gota Morota: Validation; Writing – review & editing. Hao Cheng: Conceptualization; Formal analysis; Funding acquisition; Investigation; Methodology; Project administration; Resources; Software; Supervision; Validation; Visualization; Writing – original draft; Writing – review & editing.

## CONFLICT OF INTEREST

The authors declare that the research was conducted in the absence of any commercial or financial relationships that could be construed as a potential conflict of interest.

## Supporting information

Table S1 DIC values and estimated heterogeneous residual variances for RR‐BayesC using different regression functions.
Figure S1 Window posterior probability of association (WPPA) of genomic windows at day 20 for PSA (first row) and WU (second row) in real data analysis.
Figure S2 Genomic prediction accuracy of RR‐BayesC for projected shoot area (PSA) under CV1 using different B‐spline functions as the regression functions for additive genetic effects and permanent environmental effects.
Figure S3 PSRF diagnostic plots for heterogeneous residual variances at day 1, day 6, day 11 and day 16.
Figure S4 Trace plots of three MCMC chains for genetic variances of additive genetic random regression coefficients.
