# Supplementary material for: A Bayesian random regression method using mixture priors for genome‐enabled analysis of time‐series high‐throughput phenotyping data
Source: Plant Genome. 2022 Jul 29;15(3):e20228. doi: 10.1002/tpg2.20228 (PMC12807193; doi:10.1002/tpg2.20228)
Supplement: Supplementary file 1 [file TPG2-15-e20228-s001.pdf]

Supplemental Material

Joint Gibbs sampler for random regression model using BayesCΠ

In this section, equation 5 has been written as below for simplicity,

$$\begin{aligned}\mathbf{y}_i &= \boldsymbol{\mu} + \mathbf{Z}_{a_i(t)} \sum_{j=1}^{p} m_{ij}\boldsymbol{\alpha}_j + \mathbf{e}_i \\ &= \boldsymbol{\mu} + \mathbf{Z}_{a_i(t)} \sum_{j=1}^{p} m_{ij}\mathbf{D}_j\boldsymbol{\beta}_j + \mathbf{e}_i\end{aligned}$$

where $\boldsymbol{\mu}$ is the overall mean for the phenotypic trajectory, and the random regression of permanent environmental effects is ignored. The full conditional distribution of $\boldsymbol{\beta}_j$ (i.e., the marker effects of locus j when it has effects on all the regression coefficients) is derived as follows. Let $\mathbf{B}_{ij} = \mathbf{Z}_{a_i(t)}\mathbf{D}_j$,

$$\begin{aligned}f(\boldsymbol{\beta}_j|ELSE) &\propto exp[-\sum_{i=1}^{n} \frac{(\mathbf{w}_{ij} - m_{ij}\mathbf{B}_{ij}\boldsymbol{\beta}_j)'(\mathbf{w}_{ij} - m_{ij}\mathbf{B}_{ij}\boldsymbol{\beta}_j)}{2\sigma_e^2}] \\ &\times exp(-\frac{1}{2}\boldsymbol{\beta}_j'\mathbf{K}_\alpha^{-1}\boldsymbol{\beta}_j) \\ &\propto exp[-\frac{1}{2}\frac{\sum_{i=1}^{n}(\mathbf{w}_{ij}'\mathbf{w}_{ij} - 2m_{ij}\mathbf{w}_{ij}'\mathbf{B}_{ij}\boldsymbol{\beta}_j + m_{ij}^2\boldsymbol{\beta}_j'\mathbf{B}_{ij}'\mathbf{B}_{ij}\boldsymbol{\beta}_j)}{\sigma_e^2}] \\ &\times exp(-\frac{1}{2}\boldsymbol{\beta}_j'\mathbf{K}_\alpha^{-1}\boldsymbol{\beta}_j) \\ &\propto exp\{-\frac{1}{2}[\boldsymbol{\beta}_j'(\frac{\sum_{i=1}^{n} m_{ij}^2\mathbf{B}_{ij}'\mathbf{B}_{ij}}{\sigma_e^2} + \mathbf{K}_\alpha^{-1})\boldsymbol{\beta}_j - 2\frac{\sum_{i=1}^{n} m_{ij}\mathbf{w}_{ij}'\mathbf{B}_{ij}}{\sigma_e^2}\boldsymbol{\beta}_j]\} \\ &\propto exp\{-\frac{1}{2}[\boldsymbol{\beta}_j'\mathbf{C}_j\boldsymbol{\beta}_j - 2\mathbf{r}_j'\boldsymbol{\beta}_j]\} \\ &\propto exp\{-\frac{1}{2}(\boldsymbol{\beta}_j' - \mathbf{r}_j'\mathbf{C}_j^{-1})\mathbf{C}_j(\boldsymbol{\beta}_j - \mathbf{C}_j^{-1}\mathbf{r}_j)\} \\ &\propto N(\mathbf{C}_j^{-1}\mathbf{r}_j, \mathbf{C}_j^{-1})\end{aligned}$$

where $\mathbf{w}_{ij} = \mathbf{y}_i - \boldsymbol{\mu} - \sum_{l\neq j} m_{il}\mathbf{Z}_{a_i(t)}\mathbf{D}_l\boldsymbol{\beta}_l$, $\mathbf{C}_j = \frac{\mathbf{D}_j'(\sum_{i=1}^{n} m_{ij}^2\mathbf{Z}_{a_i(t)}'\mathbf{Z}_{a_i(t)})\mathbf{D}_j}{\sigma_e^2} + \mathbf{K}_\alpha^{-1}$ and $\mathbf{r}_j = \frac{\sum_{i=1}^{n} m_{ij}\mathbf{D}_j'\mathbf{Z}_{a_i(t)}'\mathbf{w}_{ij}}{\sigma_e^2}$.

Due to the high correlation between $\boldsymbol{\beta}_j$ and δ_j, a joint posterior distribution for $\boldsymbol{\beta}_j$ and δ_j is further derived. Here, we simply use numerical labels "1", "2", ... "l" to numerate the 2^{m_a+1} possible combinations for δ_j such that

$$f(\delta_j = ''i''|\boldsymbol{\pi}) = \pi_1 I(\delta_j = ''1'') + \pi_2 I(\delta_j = ''2'') + ... + \pi_l I(\delta_j = ''l'') \tag{9}$$

where $l = 2^{m_a+1}$ and $\sum_{i=1}^{l}\pi_i = 1$. Let $\boldsymbol{\theta}$ denote all other parameters except for $\boldsymbol{\beta}_j$ and δ_j, and

$$f(\boldsymbol{\beta}_j, \delta_j|\boldsymbol{\theta}, \mathbf{y}) = f(\delta_j|\boldsymbol{\theta}, \mathbf{y})f(\boldsymbol{\beta}_j|\delta_j, \boldsymbol{\theta}, \mathbf{y}) \tag{10}$$

Note that we have already derived the distribution of $f(\boldsymbol{\beta}_j|\delta_j, \boldsymbol{\theta}, \mathbf{y})$ above and the marginal full conditional distribution of δ_j is shown as:

$$f(\delta_j|\boldsymbol{\theta}, \mathbf{y}) = \frac{f(\delta_j, \boldsymbol{\theta}, \mathbf{y})}{\sum_{\delta_j} f(\delta_j, \boldsymbol{\theta}, \mathbf{y})} \tag{11}$$

Since $f(\delta_j, \boldsymbol{\theta}, \mathbf{y}) = f(\mathbf{y}|\boldsymbol{\theta}, \delta_j) \times f(\boldsymbol{\theta}) \times f(\delta_j|\boldsymbol{\pi})$ and $\boldsymbol{\theta}$ is not correlated to δ_j, the marginal full conditional distribution of δ_j can further be expressed as:

$$f(\delta_j|\boldsymbol{\theta}, \mathbf{y}) = \frac{f(\mathbf{y}|\boldsymbol{\theta}, \delta_j) f(\delta_j|\boldsymbol{\pi})}{\sum_{\delta_j} f(\mathbf{y}|\boldsymbol{\theta}, \delta_j) f(\delta_j|\boldsymbol{\pi})} \tag{12}$$

Denote $\mathbf{w}_{ij} = \mathbf{y}_i - \boldsymbol{\mu} - \sum_{l \neq j} m_{il} \mathbf{Z}_{a_i(t)} \mathbf{D}_l \boldsymbol{\beta}_l$ and $\mathbf{B}_{ij} = \mathbf{Z}_{a_i(t)} \mathbf{D}_j$, the distribution $f(\mathbf{y}|\boldsymbol{\theta}, \delta_j)$ required for the derivation of the marginal full conditional distribution of δ_j is shown as follows.

$$\begin{aligned}
f(\mathbf{y}|\boldsymbol{\theta}, \delta_j) &= \int f(\mathbf{y}, \boldsymbol{\beta}_j|\boldsymbol{\theta}, \delta_j) d\boldsymbol{\beta}_j \\
&= \int f(\mathbf{y}|\boldsymbol{\beta}_j, \delta_j, \boldsymbol{\theta}) f(\boldsymbol{\beta}_j|\mathbf{K}_\alpha) d\boldsymbol{\beta}_j \\
&\propto \int \{exp[-\frac{1}{2\sigma_e^2} \sum_{i=1}^{n} (\mathbf{w}_{ij} - m_{ij}\mathbf{B}_{ij}\boldsymbol{\beta}_j)'(\mathbf{w}_{ij} - m_{ij}\mathbf{B}_{ij}\boldsymbol{\beta}_j)] \\
&\times exp(-\frac{1}{2}\boldsymbol{\beta}_j'\mathbf{K}_\alpha^{-1}\boldsymbol{\beta}_j)\} d\boldsymbol{\beta}_j \\
&\propto \int \{exp[-\frac{1}{2\sigma_e^2} \sum_{i=1}^{n} (\mathbf{w}_{ij}'\mathbf{w}_{ij} - 2m_{ij}\mathbf{w}_{ij}'\mathbf{B}_{ij}\boldsymbol{\beta}_j \; + m_{ij}^2\boldsymbol{\beta}_j'\mathbf{B}_{ij}'\mathbf{B}_{ij}\boldsymbol{\beta}_j)] \\
&\times exp(-\frac{1}{2}\boldsymbol{\beta}_j'\mathbf{K}_\alpha^{-1}\boldsymbol{\beta}_j)\} d\boldsymbol{\beta}_j \\
&\propto \int exp\{-\frac{1}{2}[\, \boldsymbol{\beta}_j'\mathbf{C}_j\boldsymbol{\beta}_j - 2\mathbf{r}_j'\boldsymbol{\beta}_j + \frac{1}{\sigma_e^2}\sum_{i=1}^{n} \mathbf{w}_{ij}'\mathbf{w}_{ij}] \,\} d\boldsymbol{\beta}_j \\
&\propto \int exp\{-\frac{1}{2}(\boldsymbol{\beta}_j' - \mathbf{r}_j'\mathbf{C}_j^{-1})\mathbf{C}_j(\boldsymbol{\beta}_j - \mathbf{C}_j^{-1}\mathbf{r}_j) + \\
&\frac{1}{\sigma_e^2}\sum_{i=1}^{n} \mathbf{w}_{ij}'\mathbf{w}_{ij} - \mathbf{r}_j'\mathbf{C}_j^{-1}\mathbf{r}_j\} d\boldsymbol{\beta}_j \\
&\propto exp\{\frac{1}{\sigma_e^2}\sum_{i=1}^{n} \mathbf{w}_{ij}'\mathbf{w}_{ij} - \mathbf{r}_j'\mathbf{C}_j^{-1}\mathbf{r}_j\} \\
&\times \int exp\{-\frac{1}{2}(\boldsymbol{\beta}_j' - \mathbf{r}_j'\mathbf{C}_j^{-1})\mathbf{C}_j(\boldsymbol{\beta}_j - \mathbf{C}_j^{-1}\mathbf{r}_j)\} d\boldsymbol{\beta}_j \\
&\propto exp\{\frac{1}{\sigma_e^2}\sum_{i=1}^{n} \mathbf{w}_{ij}'\mathbf{w}_{ij} - \mathbf{r}_j'\mathbf{C}_j^{-1}\mathbf{r}_j\} \times |C_j^{-1}|^{\frac{1}{2}} \times \\
&\int |C_j^{-1}|^{-\frac{1}{2}} exp\{-\frac{1}{2}(\boldsymbol{\beta}_j' - \mathbf{r}_j'\mathbf{C}_j^{-1})\mathbf{C}_j(\boldsymbol{\beta}_j - \mathbf{C}_j^{-1}\mathbf{r}_j)\} d\boldsymbol{\beta}_j \\
&\propto |C_j^{-1}|^{\frac{1}{2}} \times exp\{\frac{1}{\sigma_e^2}\sum_{i=1}^{n} \mathbf{w}_{ij}'\mathbf{w}_{ij} - \mathbf{r}_j'\mathbf{C}_j^{-1}\mathbf{r}_j\}
\end{aligned}$$

where $\mathbf{C}_j = \frac{\mathbf{D}_j'(\sum_{i=1}^{n} m_{ij}^2 \mathbf{Z}_{a_i(t)}' \mathbf{Z}_{a_i(t)})\mathbf{D}_j}{\sigma_e^2} + \mathbf{K}_\alpha^{-1}$ and $\mathbf{r}_j = \frac{\sum_{i=1}^{n} m_{ij}\mathbf{D}_j'\mathbf{Z}_{a_i(t)}'\mathbf{w}_{ij}}{\sigma_e^2}$. Since $|C_j^{-1}|^{-\frac{1}{2}} exp\{-\frac{1}{2}(\boldsymbol{\beta}_j' - \mathbf{r}_j'\mathbf{C}_j^{-1})\mathbf{C}_j(\boldsymbol{\beta}_j - \mathbf{C}_j^{-1}\mathbf{r}_j)\}$ is the kernel for normal distribution $N(\mathbf{C}_j^{-1}\mathbf{r}_j, \mathbf{C}_j^{-1})$, $\int |C_j^{-1}|^{-\frac{1}{2}} exp\{-\frac{1}{2}(\boldsymbol{\beta}_j' - \mathbf{r}_j'\mathbf{C}_j^{-1})\mathbf{C}_j(\boldsymbol{\beta}_j - \mathbf{C}_j^{-1}\mathbf{r}_j)\} d\boldsymbol{\beta}_j$ equals a constant.

Given Equ.12, the marginal full conditional distribution of δ_j is

$$f(\delta_j = "i"|\boldsymbol{\theta}, \mathbf{y}) = \frac{\pi_i \times |C_j^{-1}|^{\frac{1}{2}} \times exp\{\frac{1}{\sigma_e^2}\sum_{i=1}^{n} \mathbf{w}'_{ij}\mathbf{w}_{ij} - \mathbf{r}'_j\mathbf{C}_j^{-1}\mathbf{r}_j\}}{\sum_{\delta_j} \pi_j \times |C_j^{-1}|^{\frac{1}{2}} \times exp\{\frac{1}{\sigma_e^2}\sum_{i=1}^{n} \mathbf{w}'_{ij}\mathbf{w}_{ij} - \mathbf{r}'_j\mathbf{C}_j^{-1}\mathbf{r}_j\}}$$
$$= \frac{\pi_i \times |C_j^{-1}|^{\frac{1}{2}} \times exp\{\mathbf{r}'_j\mathbf{C}_j^{-1}\mathbf{r}_j\}}{\sum_{\delta_j} \pi_j \times |C_j^{-1}|^{\frac{1}{2}} \times exp\{\mathbf{r}'_j\mathbf{C}_j^{-1}\mathbf{r}_j\}}\}$$

Simulation of Individual Temporal Additive Genetic Effects

Based on the simulated QTL positions and QTL effects, individual random regression coefficients of additive genetic effects were computed as follows.

$$\mathbf{a}_{\cdot 0} = \mathbf{M}_{QTL,0}\boldsymbol{\alpha}_{0,QTL}$$
$$\mathbf{a}_{\cdot 1} = \mathbf{M}_{QTL,1}\boldsymbol{\alpha}_{1,QTL}$$
$$\mathbf{a}_{\cdot 2} = \mathbf{M}_{QTL,2}\boldsymbol{\alpha}_{2,QTL}$$

where $\mathbf{a}_{\cdot l}$ is a vector of length n of the $(l+1)$th random regression coefficients of additive genetic effects, $\mathbf{M}_{QTL,l}$ is an $n \times 20$ sub-matrix of marker covariates allocating the $(l+1)$th random regression coefficients to the corresponding marker effects $\boldsymbol{\alpha}_{l,QTL}$. Let $\mathbf{Z}_{a_i}$ be a 20×3 matrix of regression covariates, the breeding values (i.e., additive genetic effects) of individual i at the 20 time points were computed as

$$\begin{bmatrix} BV_{i,1} \\ \vdots \\ BV_{i,20} \end{bmatrix} = \mathbf{Z}_{a_i} \times \begin{bmatrix} \mathrm{a}_{i0} \\ \mathrm{a}_{i1} \\ \mathrm{a}_{i2} \end{bmatrix}$$

Model Specification

Single-Trait BayesC Model (ST-BayesC)

$$\mathbf{y}_t = \mathbf{1}\mu + \mathbf{M}\boldsymbol{\alpha} + \mathbf{e}, \tag{13}$$

where $\mathbf{y}_t$ is a vector of phenotypes observed at time t, μ is the intercept, $\mathbf{M}$ is a matrix of marker covariates, $\boldsymbol{\alpha}$ is a vector of marker effects, and $\mathbf{e}$ is a vector of residual with $\mathbf{e} \sim N(\mathbf{0}, \mathbf{I}\sigma_e^2)$. A mixture of a point mass at zero and a normal distribution is used as the distribution of markers. That is, for marker j, its distribution is expressed as

$$\alpha_j = \begin{cases} N(0, \sigma_\alpha^2) & \text{probability } (1-\pi) \\ 0 & \text{probability } \pi \end{cases} \tag{14}$$

Single-Trait GBLUP Model (ST-GBLUP)

$$\mathbf{y}_t = \mathbf{1}\mu + \mathbf{Z}\mathbf{g} + \mathbf{e} \tag{15}$$

where $\mathbf{y}_t$ is a vector of phenotypes observed at time t, μ is the intercept, $\mathbf{Z}$ is an incidence matrix allocating phenotypes to the individual additive genetic effects $\mathbf{g}$ with $\mathbf{g} \sim N(\mathbf{0}, \mathbf{G}\sigma_g^2)$, and $\mathbf{e}$ is a vector of residuals with $\mathbf{e} \sim N(\mathbf{0}, \mathbf{I}\sigma_e^2)$.

Repeatability Model In repeatability model, observations at different time points are treated as repeated measurements without considering the impact of temporal covariates. The repeatability model is written as follows.

$$\mathbf{y} = \mathbf{1}\mu + \mathbf{Z}_g\mathbf{g} + \mathbf{Z}_p\mathbf{p} + \mathbf{e}$$

where $\mathbf{y}$ is a vector of phenotypes observed across the 20 time points, μ is the general intercept, $\mathbf{g}$ is a vector of individual additive genetic effects, $\mathbf{p}$ is a vector of permanent environmental effects with $\mathbf{p} \sim N(\mathbf{0}, \mathbf{I}_n\sigma^2_{pe})$, and $\mathbf{e}$ is a vector of residuals with $\mathbf{e} \sim N(\mathbf{0}, \mathbf{I}_{nobs}\sigma^2_e)$. $\mathbf{Z}_g$ and $\mathbf{Z}_p$ are the incidence matrices allocating records to the additive genetic effects $\mathbf{g}$ and permanent environmental effects $\mathbf{p}$, respectively.

Correlation Matrix of Random Regression Coefficients in Real Data Analysis

$$Cor_{PSA} = \begin{bmatrix} 1.000 & 0.994 & 0.979 \\ 0.994 & 1.000 & 0.992 \\ 0.979 & 0.992 & 1.000 \end{bmatrix}$$

$$Cor_{WU} = \begin{bmatrix} 1.000 & 0.988 & 0.945 \\ 0.988 & 1.000 & 0.976 \\ 0.945 & 0.976 & 1.000 \end{bmatrix}$$

Model Comparison for RR-BayesC with Different Regression Functions in Real Data Analysis of PSA

Table S1 DIC values and estimated heterogeneous residual variances for RR-BayesC using different regression functions.

Model	DIC	Estimated heterogeneous residual variance matrix ($\times 10^8$)
Quadratic Legendre polynomial	145118	[0.56, 0.13,0.04, 0.05, 0.08, 0.06, 0.08, 0.07, 0.08, 0.10, 0.19, 0.34, 0.43, 0.36, 0.23, 0.37, 1.43, 5.95, 11.31, 20.52]
Linear B-splines with 1 equidistant knot	149566	[0.41, 0.10, 0.04, 0.08, 0.12, 0.07, 0.06, 0.43, 1.37, 4.14, 1.10, 0.15, 0.40, 0.63, 0.38, 0.32, 1.67, 9.34, 21.89, 45.76]
Linear B-spline with 2 equidistant knots	143285	[0.10, 0.04, 0.05, 0.06, 0.05, 0.07, 0.42, 0.09, 0.13, 0.16, 0.14, 0.12, 0.61, 2.66, 0.59, 0.55, 0.86, 0.48, 0.70, 2.21]
Quadratic B-spline with 1 equidistant knot	139613	[0.05, 0.04, 0.04, 0.05, 0.05, 0.05, 0.07, 0.06, 0.08, 0.14, 0.19, 0.14, 0.16, 0.21, 0.28, 0.51, 0.50, 0.52, 0.66, 1.16]

Window-based Association Inference for Real Rice Data

When WPPA of genomic windows are used for association inference, both ST-BayesC and RR-BayesC do not infer any significant windows that passed the threshold at WPPA = 0.85 (Figure S1). The association performance of ST-BayesC and RR-BayesC both improved at day 20, where WPPA of top windows increased, enlarging the differentiation between potentially significant windows and rest of the windows.

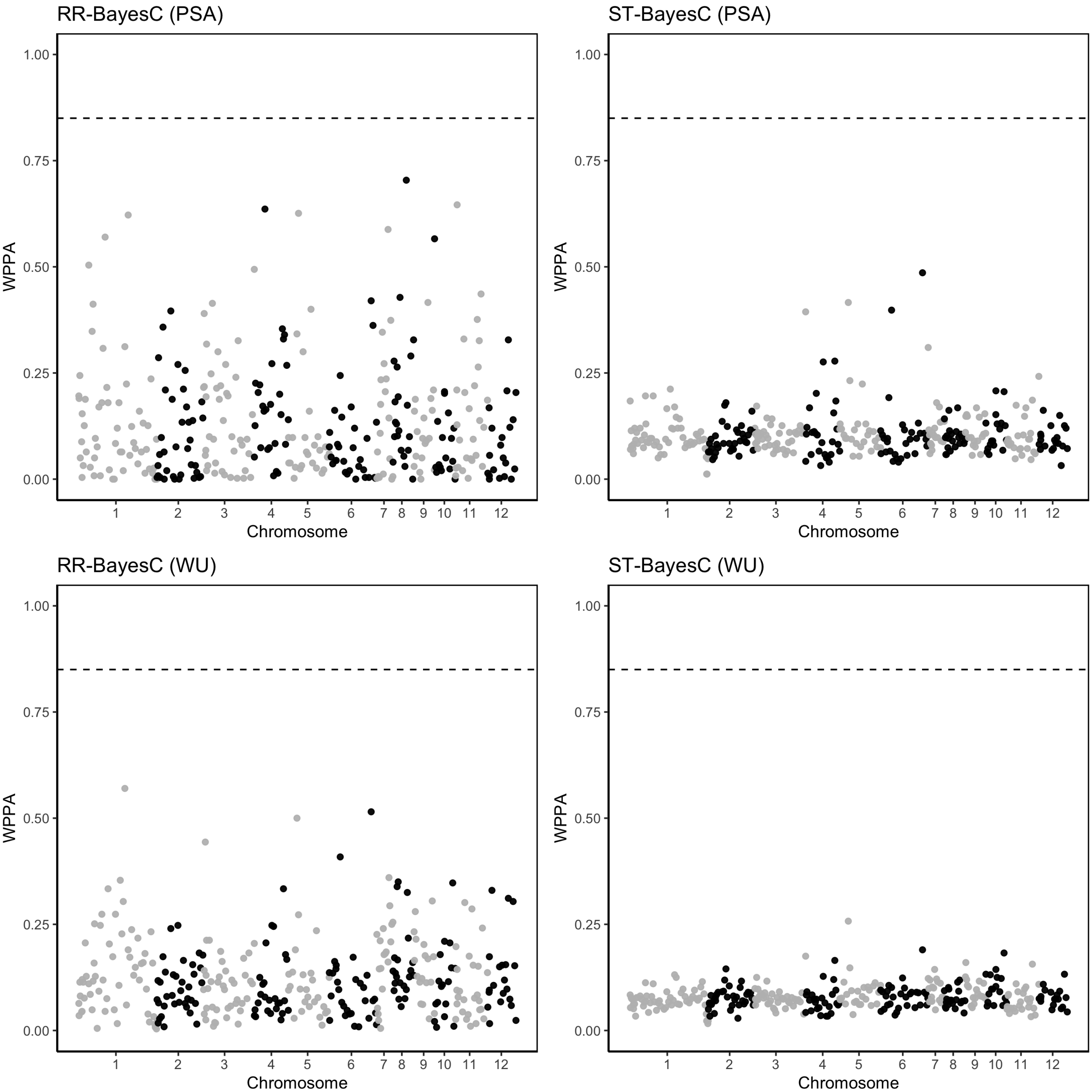

Figure S1 Window posterior probability of association (WPPA) of genomic windows at day 20 for PSA (first row) and WU (second row) in real data analysis. The performance of RR-BayesC is shown at the left hand side and the performance of ST-BayesC is shown at the right hand side.

1187 ***Genomic Prediction in CV1 using different regression functions by RR-BayesC models.***

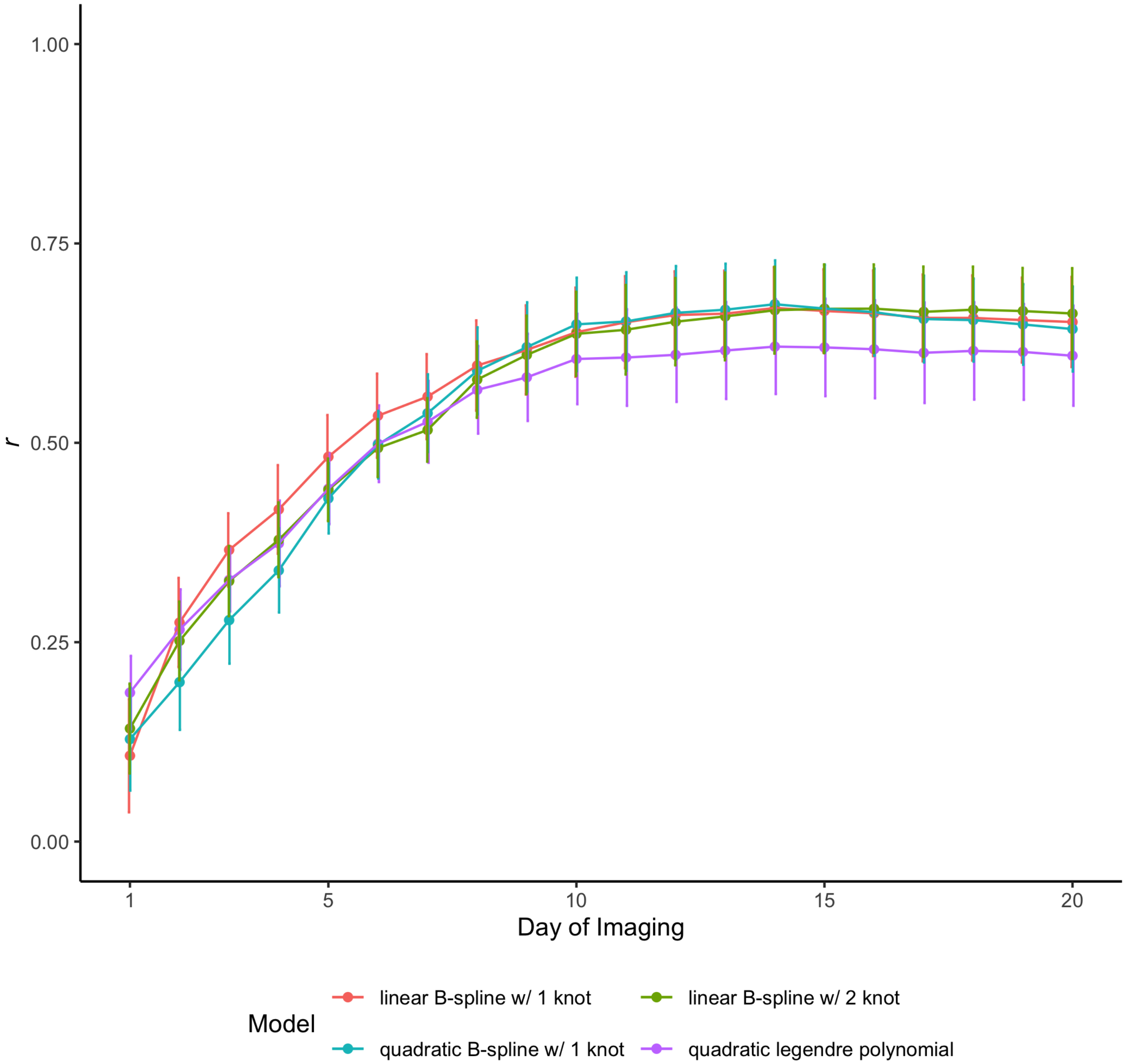

Figure S2 Genomic prediction accuracy for projected shoot area (PSA) under CV1 using different B-spline functions as the regression functions for additive genetic effects and permanent environmental effects. Three different B-spline functions were considered: linear B-spline with 1 equidistant knot, linear B-spline with 2 equidistant knot, and quadratic B-spline with 1 equidistant knot.

Diagnostic Plots for Convergence of MCMC Chains

PSRF diagnostic plots (Figure S3) for heterogeneous residual variances at day 1, day 6, day 11 and day 16 are provided to show the convergence of MCMC chains for RR-BayesC model (using Legendre polynomials). Chain length is of 100,000 with first 50,000 iterations discarded as burnin. R package "coda" (Plummer *et al.* 2006) is used to generate the diagnostic plots.

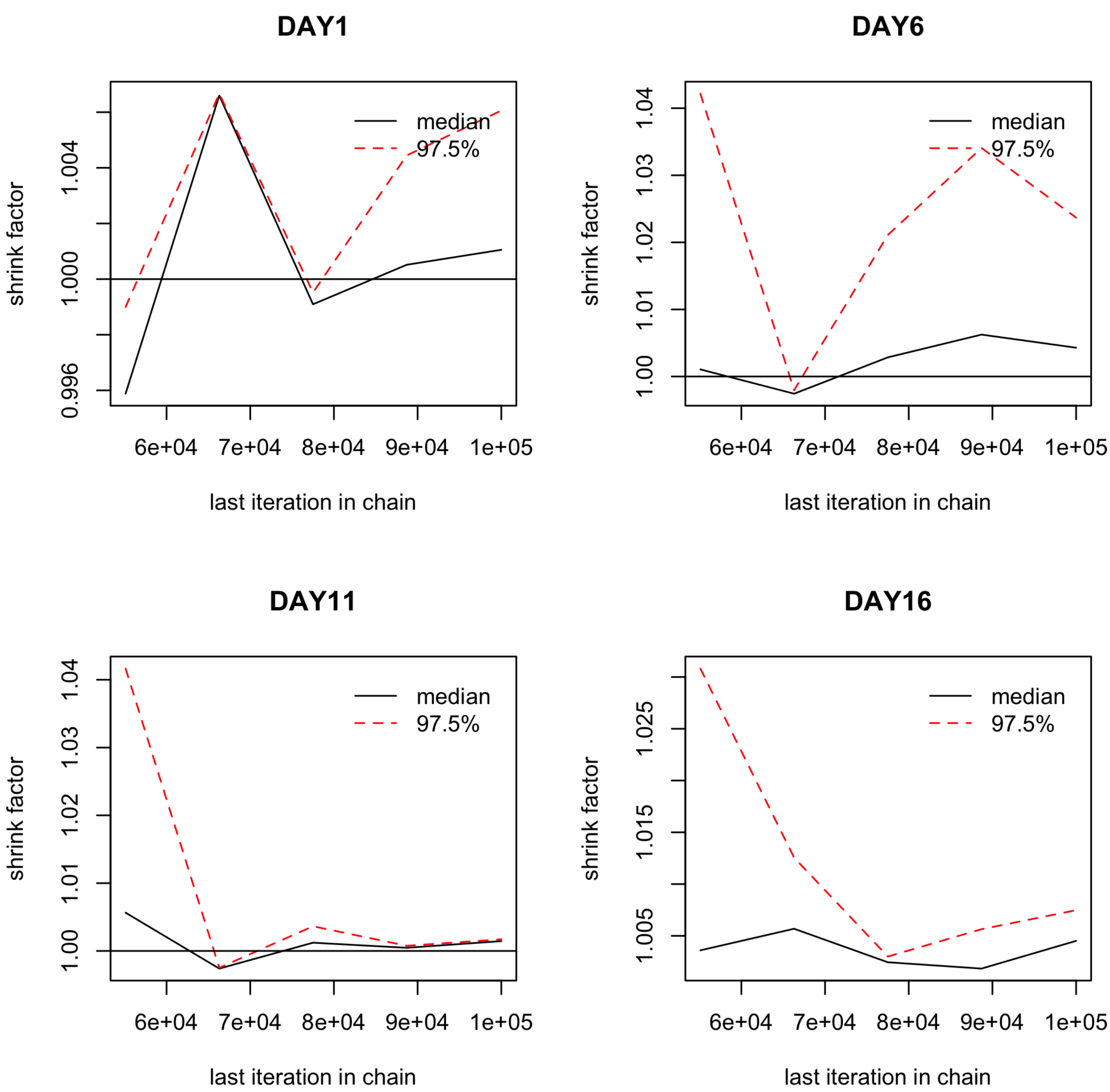

Figure S3 PSRF diagnostic plots for heterogeneous residual variances at day 1, day 6, day 11 and day 16.

Trace plots (Figure S4) for genetic variances of additive genetic random regression coefficients are provided. Three MCMC chains were considered. Chain length is of 100,000 with first 50,000 iterations discarded as burnin.

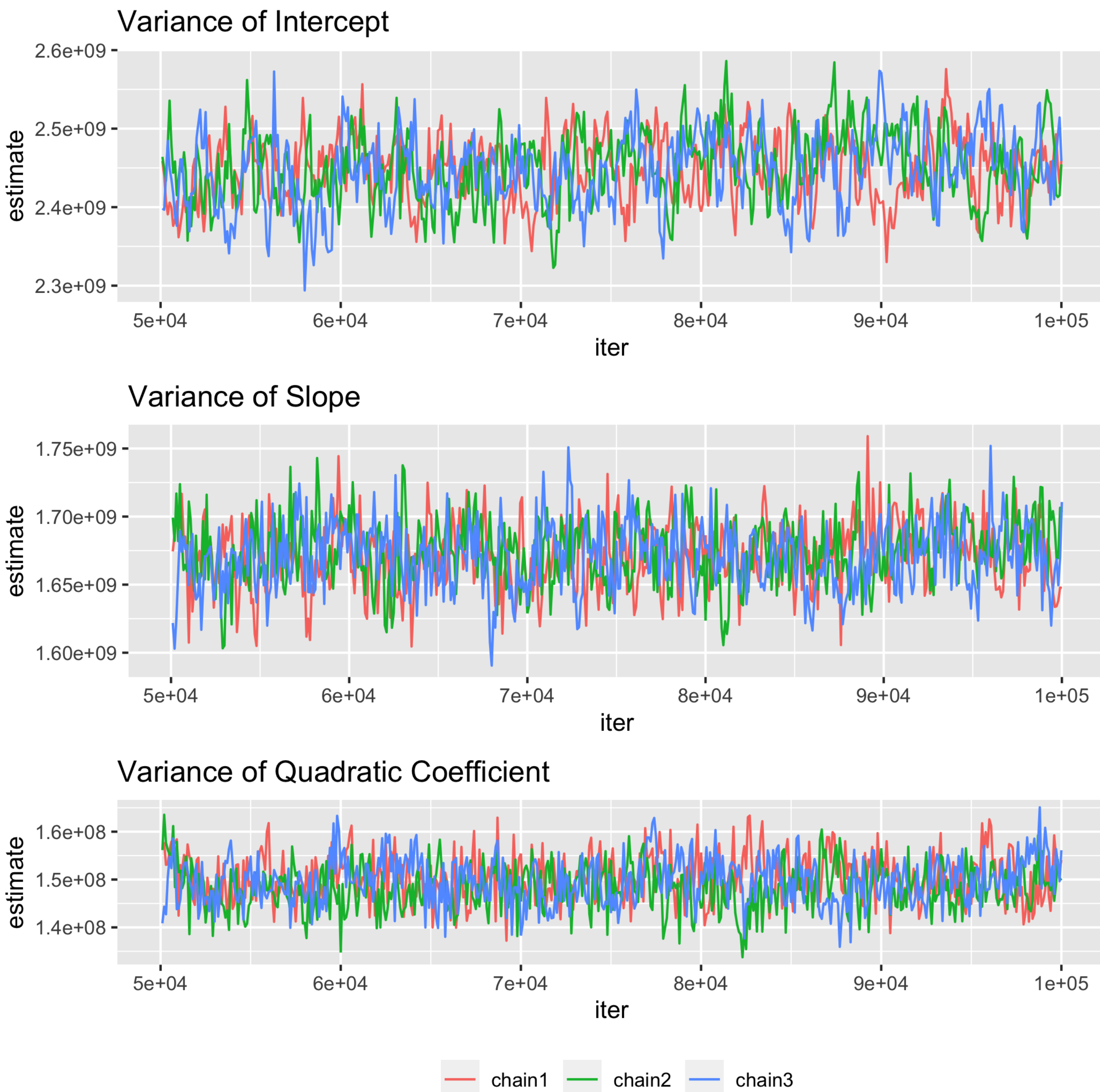

Figure S4 Trace plots of three MCMC chains for genetic variances of additive genetic random regression coefficients.
